# Supplementary material for: Expression patterns of CD24 and CD27 resolve the origins and gradual differentiation of CD11c+ atypical memory B-cells after malaria infection
Source: EMBO J. 2026 Oct 11;45(21):7468–88. doi: 10.1038/s44318-026-00941-7 (PMC13728153; doi:10.1038/s44318-026-00941-7)
Supplement: Supplementary file 1 [file 44318_2026_941_MOESM1_ESM.pdf]

Appendix for

Expression patterns of CD24 and CD27 resolve the origins and gradual differentiation of CD11c+ atypical memory B-cells after malaria infection

Contents

Appendix Table S1. Descriptive statistics of the study participants. 2

Appendix Table S2. Antibody list for flow cytometry and cell sorting panels 4

Appendix Figure S1. Diversity of atypical B cell-associated markers on B cells after Hantavirus infection. 5

Appendix Figure S2. Longitudinal single-cell transcriptomic and CITE-seq analysis after acute malaria.. 6

Appendix Figure S3. Differential expression and pseudotime analysis of longitudinal single-cell transcriptomic and CITE-seq analysis after acute malaria. 7

Appendix Figure S4. Temporal kinetics of B cell populations up to one year after acute malaria.. 8

Appendix Figure S5. Differential expression analysis of 10X genomics generated single cell transcriptomic data at two weeks after acute malaria. 9

Appendix Figure S6. V-gene usage in CD11c+ B cell clusters. 10

Appendix Figure S7. B cell expression after in vitro stimulation or ex vivo after acute malaria.. 11

Appendix Figure S8. Comparison of B cell populations in matched tonsil and PBMC samples. 12

Appendix Figure S9. Functional characterization of ABC subsets............................... 13

Appendix Table S1. Descriptive statistics of the study participants.

**All donors**	**Malaria**	**Healthy**
Number of participants	60	14
Female sex, n (%)	16 (42.7)	10 (71)
Age, years, median (range)	38 (20-78)	32 (21-57)
Parasitemia, % infected RBCs, median (range)[2]	0.7 (0.009-17.0)	–
Plasmodium falciparum infection, n (%)	57 (95)	–
Previous malaria exposure, n (%)*	36 (65)	0 (0)
Related to Figure 1 (flow cytometry)		
Number of participants	4	–
Female sex, n (%)	3 (75)	–
Age, years, median (range)	47 (36-51)	–
Parasitemia, % infected RBCs, median (range)[2]	1 (0.9-1.4)	–
Plasmodium falciparum infection, n (%)	4 (100)	–
Previous malaria exposure, n (%)	3 (75)	–
Related to Figure 2 (flow cytometry)		
Number of participants	18	–
Female sex, n (%)	4 (22)	–
Age, years, median (range)	34 (27-54)	–
Parasitemia, % infected RBCs, median (range)[2]	0.4 (0.09-8)	–
Plasmodium falciparum infection, n (%)	18 (100)	–
Previous malaria exposure, n (%)	13 (72)	–
Related to Figure 2 (BD Rhapsody)		
Number of participants	4	–
Female sex, n (%)	0 (0)	–
Age, years, median (range)	42 (26-62)	–
Parasitemia, % infected RBCs, median (range)[2]	0.7 (0.1-1.4)	–
Plasmodium falciparum infection, n (%)	4 (100)	–
Previous malaria exposure, n (%)	2 (50)	–
Related to Figure 3/4/5 (flow cytometry + 10X)		
Number of participants	9	6
Female sex, n (%)	5 (56)	5 (83.3)
Age, years, median (range)	46 (22-78)	32 (24-46)
Parasitemia, % infected RBCs, median (range)[2]	1.2 (0.1-7.0)	–
Plasmodium falciparum infection, n (%)	6 (67)	–
Previous malaria exposure, n (%)*	–	0 (0)
Related to Appendix Figure S4		
Number of participants	51	14
Female sex, n (%)	11 (22)	10 (71)

Age, years, median (range)	37 (20-68)	32 (21-57)
Parasitemia, % infected RBCs, median (range)[2]	0.6 (0.009-17)	–
Plasmodium falciparum infection, n (%)	51 (100)	–
Origin in malaria endemic area, n (%)	33 (65)	0 (0)

*Missing information from 5 donors

Appendix Table S2. Antibody list for flow cytometry and cell sorting panels

Antibody specific for	Clone	Fluorochromes	Company
CD1c	F10/21A3	BB515	BD
CD3	UCHT1	APC-R700	BD
CD4	SK3	BV750	BD
CD8	SK1	PerCp-Cy5.5	BD
CD10	HI10a	APC-R700	BD
CD11c	B-ly6	BB515, BV650	BD
CD19	HIB19	PE-Cy7, APC-R700	BD
CD19	SJ25C1	BUV737	BD
CD20	2H7	APC-H7, BV510	BD
CD24	ML5	PE-CF594, PE	BD
CD21	HB5	PE-Cy7	ThermoFisher Scientific
CD21	B-ly4	BV421	BD
CD27	M-T271	BV650, BV786	BD
CD38	HIT2	PerCp-Cy5.5	BD
CD38	HIT2	APC-Fire750, APC-Fire810	Biolegend
CD55 (DAF)	IA10	BB515	BD
CD56	NCAM16.2	APC-R700	BD
CD62L	DREG-56	BV480	BD
CD80	B7-1	PE-Cy5	Thermo Fisher Scientific
CD86	IT2.2	PE-Cy5	Biolegend
CD85j	GHI/75	Biotin	BD
CD268/BAFF-R	11C1	Alexa Fluor 647	BD
CCR1	53504R	PE-Cy5.5	Novus
CXCR3	1C6	PE-Cy5, PE-Cy7	BD
FcRL3	H5/FcRL3	Alexa Fluor 647	Biolegend
FcRL4	A1	BUV661	BD
FcRL5	509f6	PE/RB613	Biolegend
Ki67	B56	BV421	BD
IgD	IA6-2	BB515, BB700, FITC	BD
IgM	G20-127	BV605	BD
IL10	JES3-9D7	R718	BD
NKG7	2G9A10F5	PE	Beckman Coulter
T-bet	O4-46	Alexa Fluor 647	BD
TNF-alpha	MAb11	BV650	BD
Cell trace violet			Thermo Fisher Scientific
Live/dead		Blue, Green	Thermo Fisher Scientific
Streptavidin		BUV395	BD

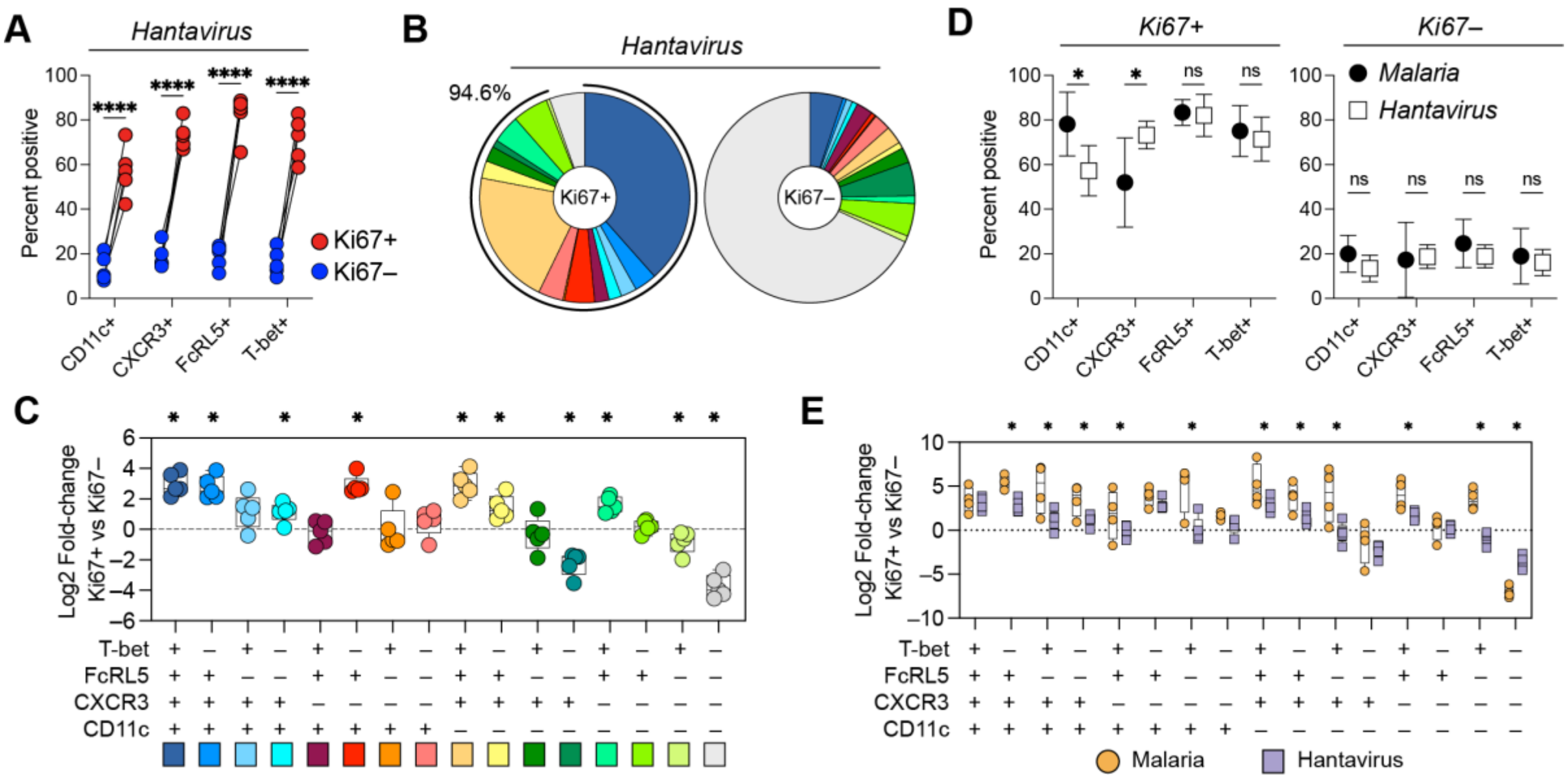

Appendix Figure S1. Diversity of atypical B cell-associated markers on B cells after Hantavirus infection. *(**A**) Frequency of mature B cells positive for CD11c, T-bet, CXCR3, and FcRL5 among Ki67+ and Ki67– cells in patients with the hantavirus PUUV. Statistics was evaluated using a paired two-way ANOV followed by Sidak's post-hoc test, ****p<0.0001. (**B**) Donut plot showing the frequencies of populations expressing one or several ABC-associated markers among Ki67+ (left) or Ki67– (right) cells in Hantavirus infection (n=5). (**C**) Log2 fold change representing the expansion of each population between Ki67+ and Ki67– cells in individuals with Hantavirus infection. Statistics evaluated using ratio paired t-tests between KI67+ and KI67– frequencies for each marker combination prior to fold-change calculation. *p<0.05. (**D**) Average +/– SD of mature B cells expressing atypical markers between participants with malaria (n=4, filled circle) and hantavirus (n=5, open boxes). Statistics was evaluated using two-way ANOVA followed by Sidak's post-hoc test for comparing specific markers. ns=p>0.05, *p<0.05. (**E**) Log$_2$ fold-change (Ki67$^+$ vs Ki67$^-$) for each marker combination, compared between malaria (orange circles) and hantavirus (purple boxes). Differences were tested by two-way ANOVA with FDR correction; *p<0.05. Source Data for this Figure could not be made freely accessible but have been deposited to the controlled-access repository Zenodo.*

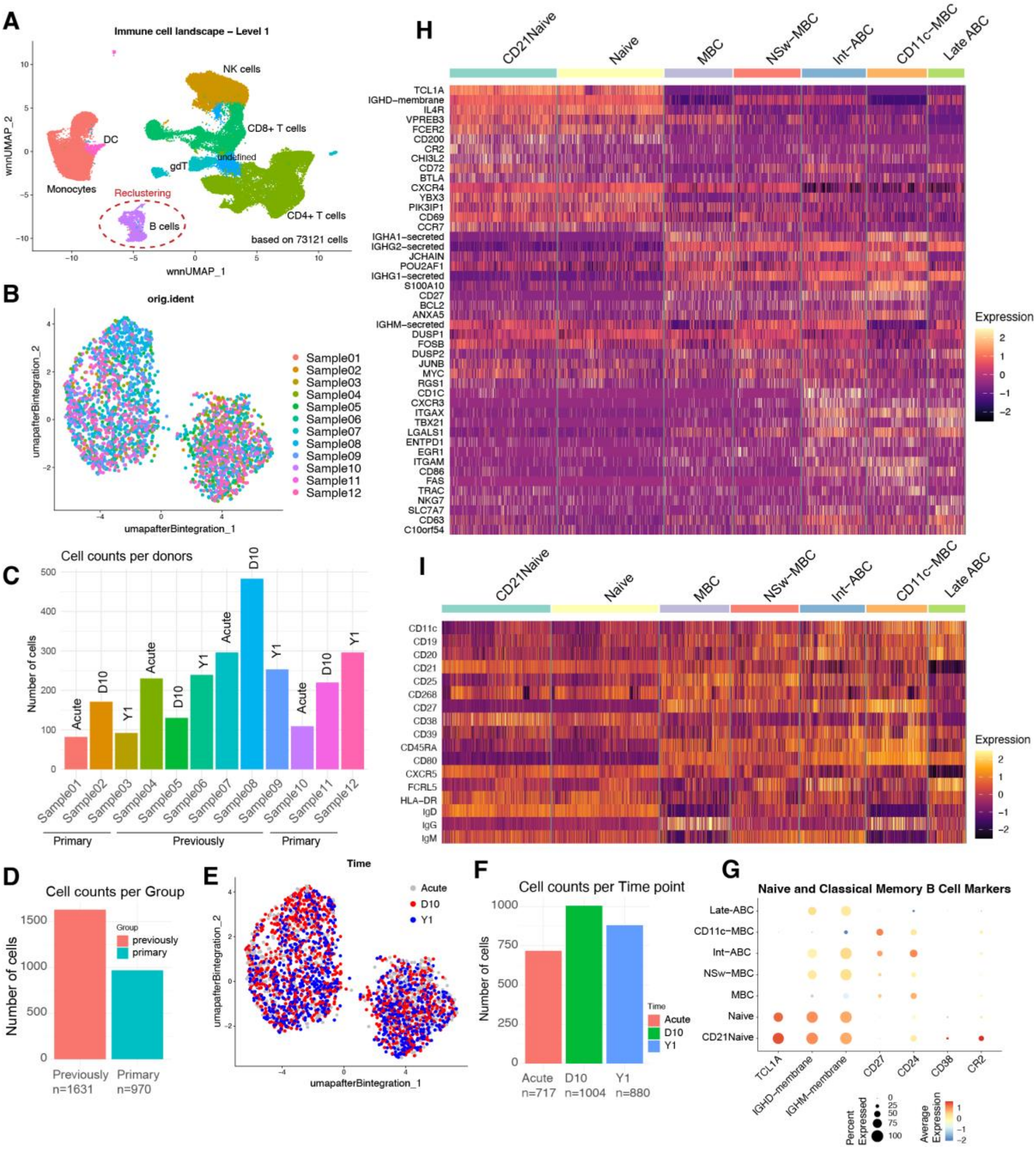

Appendix Figure S2. Longitudinal single-cell transcriptomic and CITE-seq analysis after acute malaria. ***(A)*** *UMAP clustering of all immune cells from longitudinal samples collected from malaria-infected patients.* ***(B)*** *UMAP re-clustering of the B cell compartment, colored by patient of origin.* ***(C)*** *The number of cells for each donor and time-point.* ***(D)*** *The number of cells for previously exposed and primary infected individuals.* ***(E)*** *UMAP re-clustering of the B cell compartment, colored by sampling time point (acute, 10 days post-infection, and 1-year post-infection).* ***(F)*** *The number of cells at each time-point.* ***(G)*** *Dot plot showing the expression of naive and memory B cell markers across all B cell clusters.* ***(H)*** *Heatmap of the top differentially upregulated genes across B cell clusters.* ***(I)*** *Heatmap of surface protein marker expression across all B cell clusters. Source Data for this Figure could not be made freely accessible but have been deposited to the controlled-access repository Zenodo.*

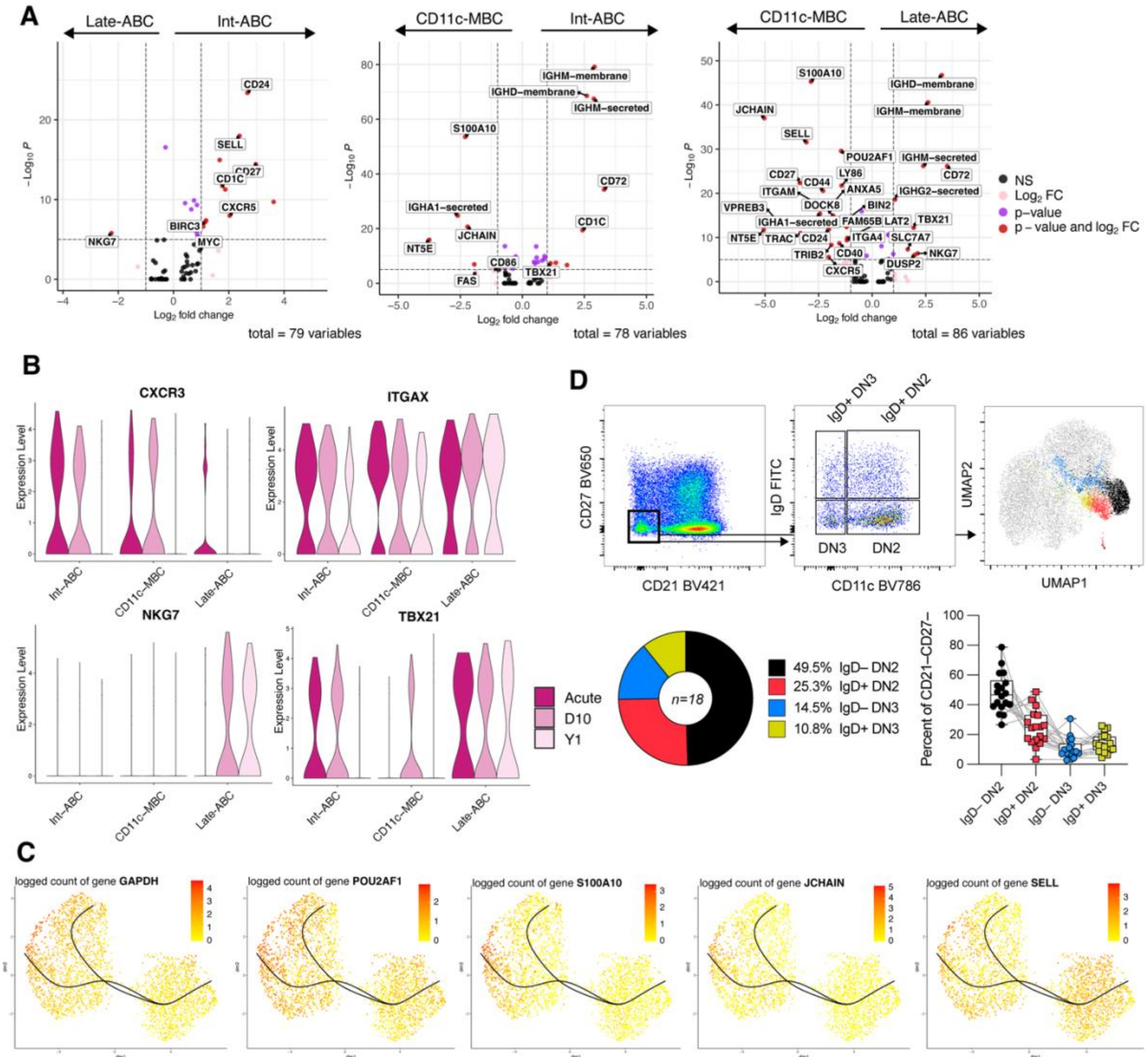

Appendix Figure S3. Differential expression and pseudotime analysis of longitudinal single-cell transcriptomic and CITE-seq analysis after acute malaria.

(A) *Volcano plot comparing the three atypical-like clusters (CD11c+ MBC, Int-ABC, and Late-ABC), highlighting differentially expressed genes.* ***(B)*** *Violin plot showing the expression of atypical B cell markers over time in the atypical-like clusters.* ***(C)*** *UMAP colored by Slingshot pseudotime, with smoothed curves representing two inferred lineages and the top five differentially expressed genes at each lineage endpoint. (**D**) Top panels indicate flow cytometry of 63,000 mature B cells concatenated from 18 donors with acute malaria. Gating for CD21– CD27– Late-ABC followed by gating for IgD versus CD11c to identify DN2 and DN3 cells (+/– IgD expression) with populations overlayed in different colors on the full UMAP embedding. Bottom panels indicate the overall (pie-graph) and individual donor (box-plots) frequency of each DN population among Late-ABCs. Source Data for this Figure could not be made freely accessible but have been deposited to the controlled-access repository Zenodo.*

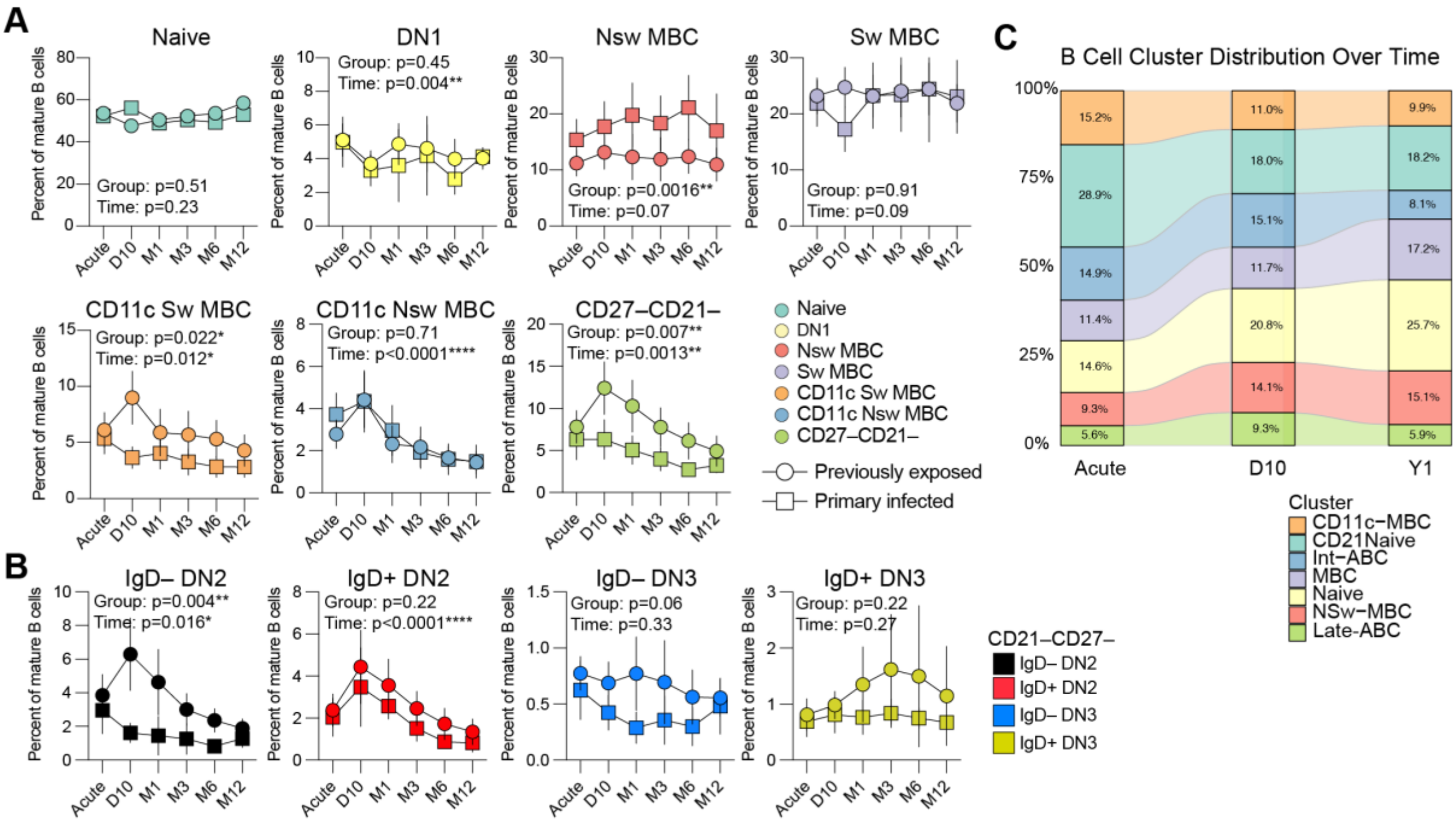

Appendix Figure S4. Temporal kinetics of B cell populations up to one year after acute malaria.

*(**A**) The frequency of Naïve (IgD+CD21+CD27–), DN1 (IgD–CD27–CD21+), Nsw MBC (IgD+CD27+), Sw MBC (IgD–CD27+), CD11c Sw MBC (IgD–CD27+CD11c+), CD11c Nsw MBC (IgD+CD27+CD11c+), and CD27–CD21– B cell subsets was assessed a the time of acute malaria (n=49), and 10 days (n=32), 1 month (n=23), 3 months (n=26), 6 months (n=23), and 12 months (n=27) after treatment initiation. Kinetics for primary infected (boxes) and previously exposed (circles) donors is shown. (**B**) Same analysis as in (A) but for IgD+ and IgD– DN2 (CD27–CD21–CD11c) and DN3 (CD27–CD21–CD11c–) subsets. (**C**) Alluvial plot indicating the frequency of cells within each cluster based on CITE-seq data during Acute malaria, and 10 days, and 1 year after treatment (n=4). Statistical analysis for (A) and (B) was done using a mixed-effects model results for comparisons between primary infected and previously exposed indicated by the Group variable and changes over time indicated by the Time variable. P-values are indicated in the graphs with *p<0.05, **p<0.01, ***p<0.001, ****p<0.0001. Source Data for this Figure could not be made freely accessible but have been deposited to the controlled-access repository Zenodo.*

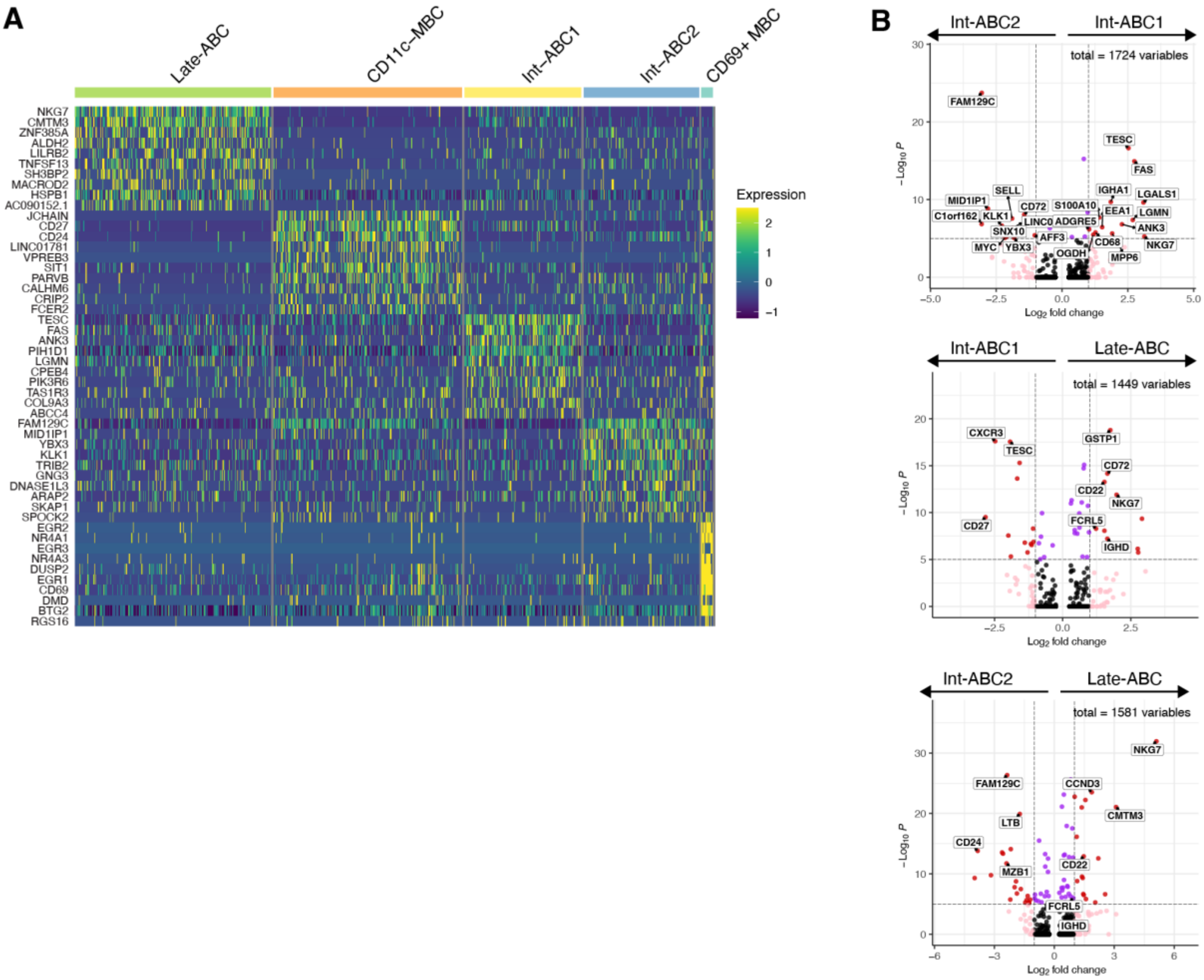

Appendix Figure S5. Differential expression analysis of 10X genomics generated single cell transcriptomic data at two weeks after acute malaria.

(A) *Heatmap of the top 10 differentially expressed genes across all CD11c+ B cell clusters.* ***(B)*** *Volcano plot comparing the three atypical-like clusters (Int-ABC1, Int-ABC2, and Late-ABC), highlighting differentially expressed genes. Source Data for this Figure could not be made freely accessible but have been deposited to the controlled-access repository Zenodo.*

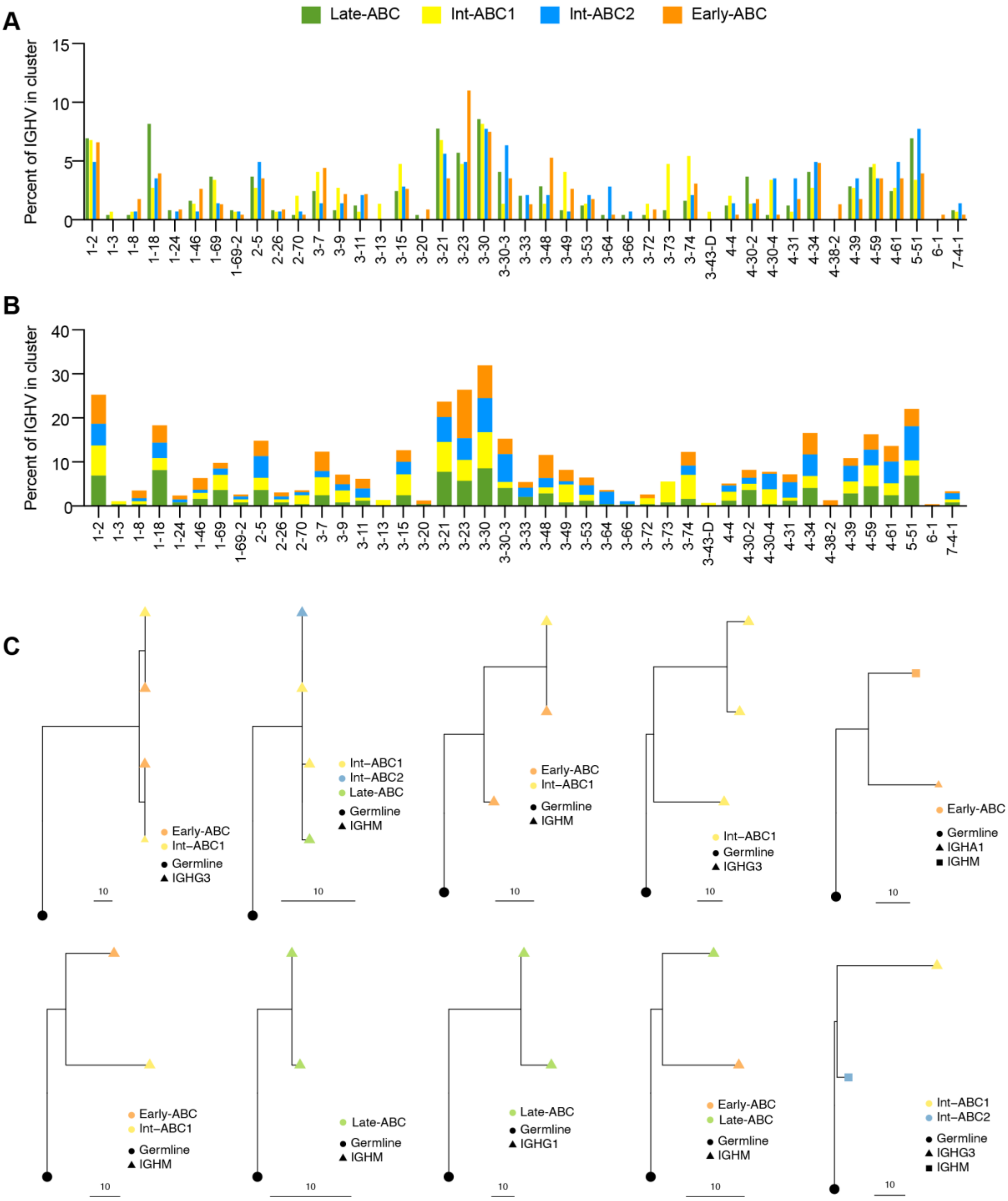

Appendix Figure S6. V-gene usage in CD11c+ B cell clusters.

*(**A-B**) The frequency of gene usage for each V-gene segment out of all sequenes for each cluster with Late-ABC (green, n=245), Int-ABC1 (yellow, n=147), Int-ABC2 (blue, n=142), and Early-ABC (orange, n=227). The frequencies are shown side by side (**A**) or summarized (**B**). (**C**) Clonal lineage trees based on single cell V(D)J sequence data. Inferred germlines are indicate by black dot. Color indicates which cluster the cells come from. The symbol indicates B cell receptor isotype with the key in the individual lineage trees. Distance is indicated by line corresponding to mutations compared with germline. Source Data for this Figure could not be made freely accessible but have been deposited to the controlled-access repository Zenodo.*

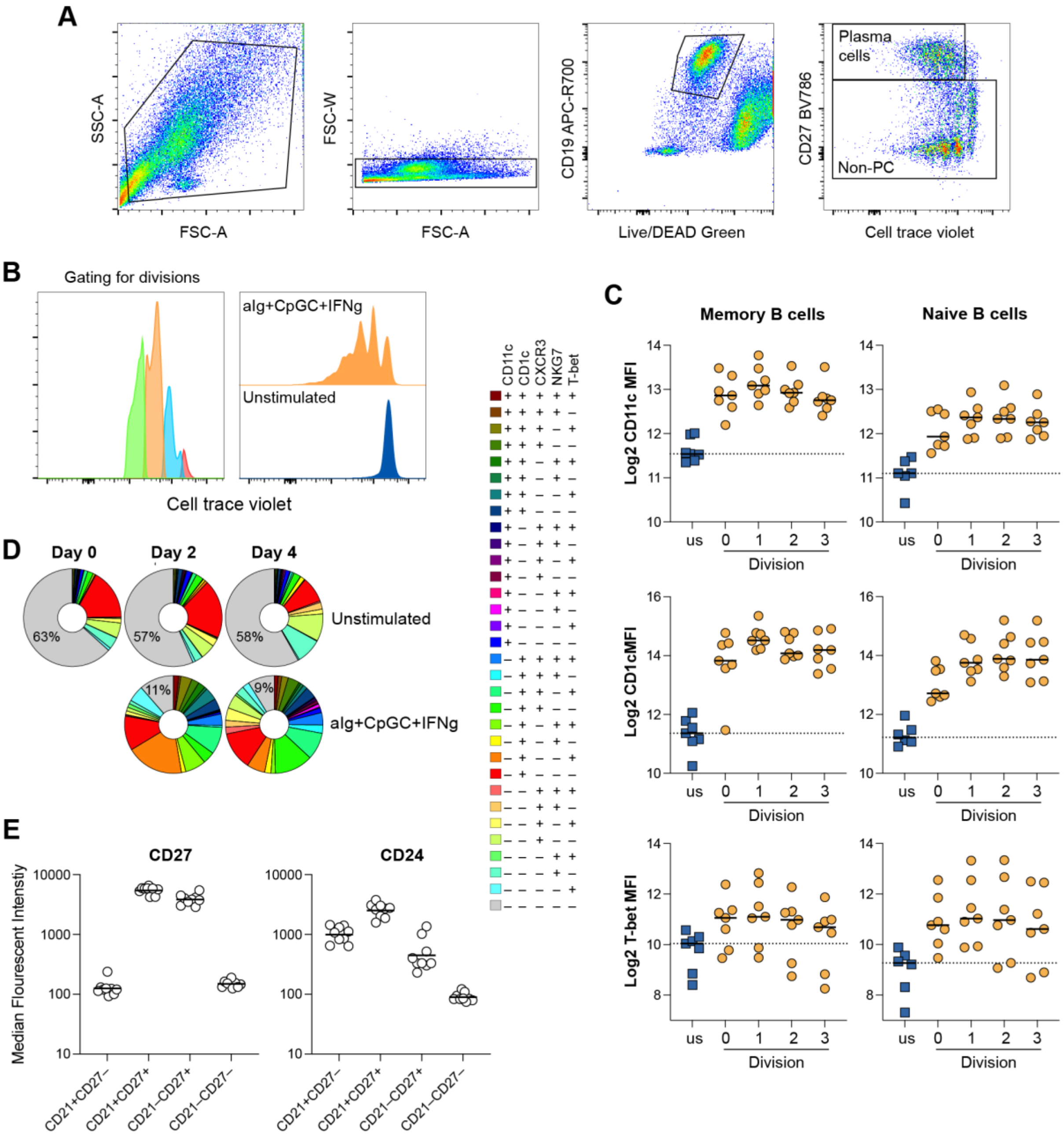

Appendix Figure S7. B cell expression after in vitro stimulation or ex vivo after acute malaria.

*(**A**) Gating strategy to analyze marker expression on non-plasma cells (PC). Example shows sorted memory cells stimualted with aIg+CpGC+IFNg for 4 days. (**B**) Gating of cells after different divisions (left) and example of cell trace violet dilution for stimulated (orange) and unstimulated (blue) cells (right). (**C**) CTV labelled sorted memory and naïve B cells left unstimulated (blue boxes) or stimulated with anti-Ig + CpG-C + IFNg (orange circles) for 4 days. CD11c, CD1c, T-bet median flourescent intensity in undivided and dividing cells with plasma cells gated away. n=6-7 donors pooled from four separate experiments. (**D**) Boolean gating of ABC markers at Day 0, 2, and 4 of stimulation. (**E**) CD27 and CD24 median flourescent intensity in populations gated for CD21 and CD27 from malaria patients (n=9 donors) sampled at the acute time point or 10 days after treatment initiation. Source Data for this Figure could not be made freely accessible but have been deposited to the controlled-access repository Zenodo.*

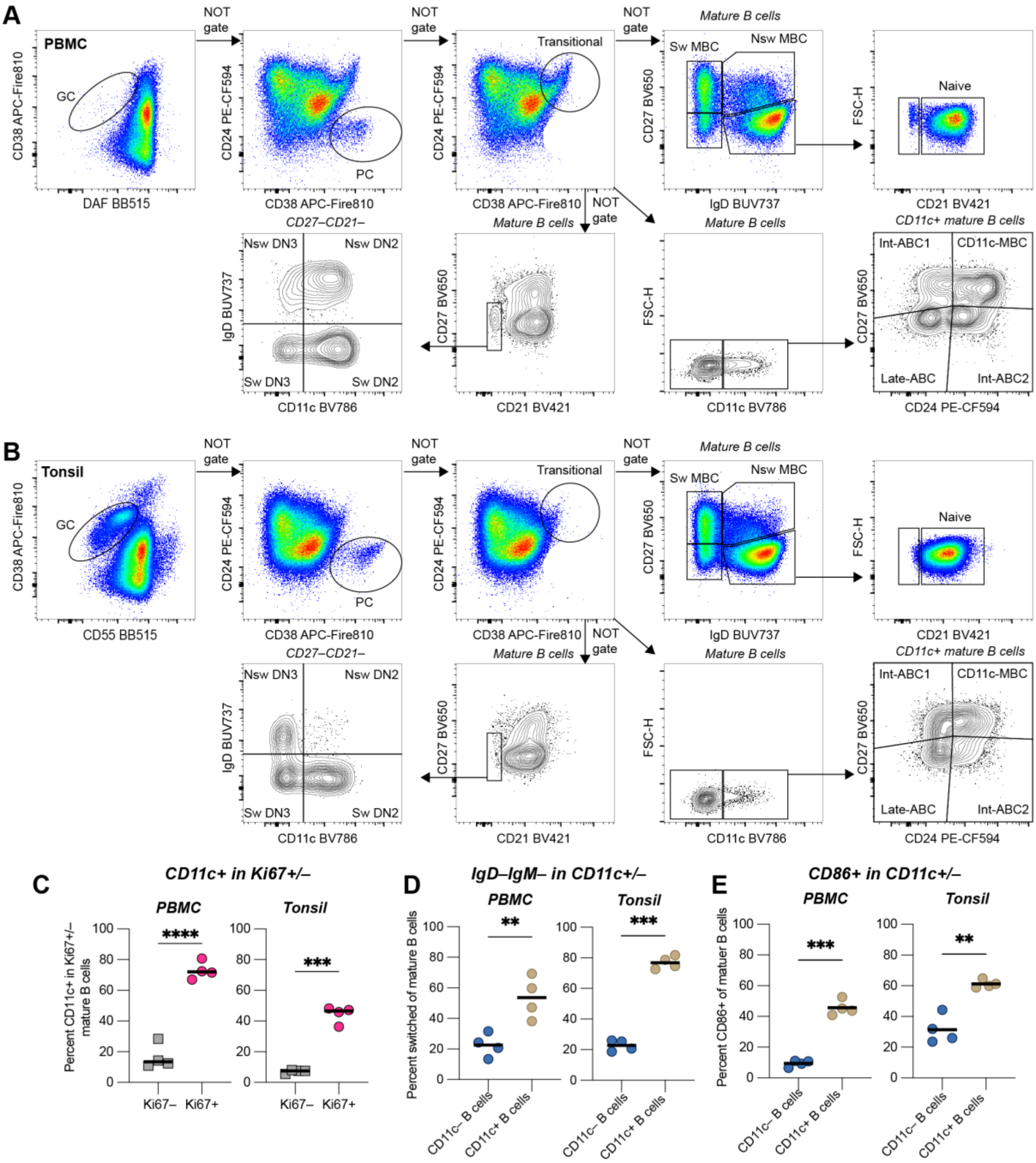

Appendix Figure S8. Comparison of B cell populations in matched tonsil and PBMC samples.
*(**A-B**) Gating strategy for cell populations in PBMC (**A**) and Tonsil (**B**) samples indicated in main Figure 6. FACS plots represent concatenated data from all 4 donors. (**C-E**) Each symbol indicates a donor. Statistical analysis was done by paired t-test with **p<0.01, ***p<0.001, ****p<0.0001. (**C**) Frequency of CD11c+ B cells among Ki67– and Ki67+ mature B cells in PBMC (left, grey box) and Tonsil (right, pink circle) samples. (**D**) Frequency of switched (IgD–IgM–) B cells among CD11c– and CD11c+ mature B cells in PBMC (left, blue circle) and Tonsil (right, yellow circle) samples. (**E**) Frequency of CD86+ B cells among CD11c– and CD11c+ mature B cells in PBMC (left, blue circle) and Tonsil (right, yellow circle) samples. Source Data for this Figure could not be made freely accessible but have been deposited to the controlled-access repository Zenodo.*

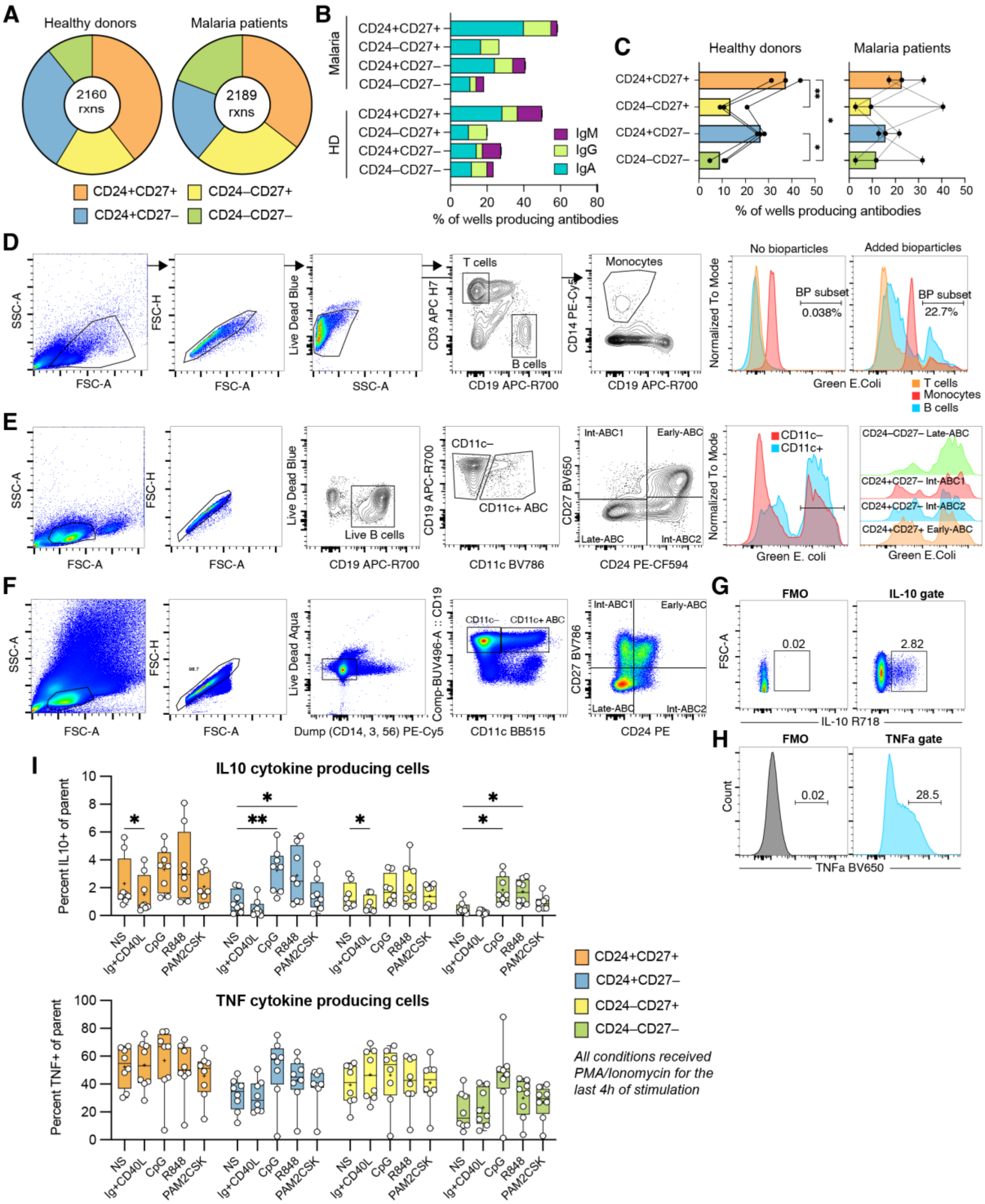

Appendix Figure S9. Functional characterization of ABC subsets.

*(**A**) Overall distribution of antibody positive wells for healthy donors (left) and malaria patients (right). (**B**) Antibody isotype contribution in the different cultured ABC populations, shown for healthy donors and malaria patients. Statistical evaluation by 2-way ANOVA followed by Tukey's posttest. *p<0.05, **p<0.01. (**C**) Comparison between ABC populations for healthy donors (left) and malaria patients (right); statistics as in (B). (**D**) Gating strategy for total T cells, B cells, and monocytes and for bioparticle positive cells. (**E**) Gating strategy for assessing Bioparticle binding in ABCs and ABC subsets. (**F**) Gating strategy for assessing intracellular cytokine production in ABCs and ABC subsets. (**G**) Fluorescence minus one (FMO) to set the gate for IL-10 and (**H**) TNF-alpha. (**I**) Percent IL10+ (top) and TNF+ (bottom) cytokine producing cells associated with indicated stimulation condition within ABC subsets. Statistical evaluation by two-tailed 2-way ANOVA followed by Dunn's posttest comparing each stimulation with non-stimulated cells (NS). Note that all cells receive PMA/Ionomycin*

for the last 4 h of culture. Only $p<0.05$ are shown. *$p<0.05$, **$p<0.01$. Source Data for this Figure could not be made freely accessible but have been deposited to the controlled-access repository Zenodo.
