# Supplementary material for: Expression patterns of CD24 and CD27 resolve the origins and gradual differentiation of CD11c+ atypical memory B-cells after malaria infection
Source: EMBO J. 2026 Oct 11;45(21):7468–88. doi: 10.1038/s44318-026-00941-7 (PMC13728153; doi:10.1038/s44318-026-00941-7)
Supplement: Supplementary file 2 [file 44318_2026_941_MOESM2_ESM.pdf]

THE EMBO JOURNAL

Expression patterns of CD24 and CD27 resolve the origins and gradual differentiation of CD11c+ atypical memory B-cells after malaria infection

Alan Dine Courey Ghaouzi, Linn Kleberg, Zaynab Mousavian, Maximilian Lautenbach, Marta Pirronello, Ganesh Phad, Mattias Forsell, Anna Färnert, and Christopher Sundling

Corresponding author(s): Christopher Sundling (christopher.sundling@ki.se)

Review Timeline:

Submission Date:	21st Oct 25
Editorial Decision:	16th Jan 26
Revision Received:	23rd Jun 26
Editorial Decision:	28th Jul 26
Revision Received:	18th Aug 26
Accepted:	11th Sep 26

Editor: Ioannis Papaioannou

Transaction Report:

(Note: With the exception of the correction of typographical or spelling errors that could be a source of ambiguity, letters and reports are not edited. Depending on transfer agreements, referee reports obtained elsewhere may or may not be included in this compilation. Referee reports are anonymous unless the Referee chooses to sign their reports.)

Dear Chris,

Thank you again for the submission of your manuscript EMBOJ-2025-121231 for consideration by The EMBO Journal, and for your patience during peer review. As I have already informed you, your manuscript has now been seen by three experts in the field, and we have received the full set of their comments, which I have shared with you (they are appended again below). I would also like to thank you for your responses to the major concerns of the referees you shared with me; they were very helpful for us to reach a well-informed and balanced decision on the manuscript.

The referees overall recognize that the manuscript presents a detailed analysis of atypical B-cells in malaria and other contexts, which provides useful and novel insights into a relevant topic. Referee #3 is very supportive pointing out that the study is well-designed, carefully performed and appropriately presented, referee #2 also acknowledges that the detailed analysis presented in this manuscript would be a valuable source of data about these cells, and referee #1 also appreciates the context of the study. However, all referees also identify limitations and raise some major concerns, regarding the weak relationship of the findings with the malaria context, the sample sizes and analyses in some parts of the study, the proposed differentiation scenario that is presented as a main conclusion of the work, and also the need for further functional experiments, among others.

On balance, after carefully considering the referees' comments and recommendations, as well as your draft response to the major raised concerns, I would like to invite you to submit a thoroughly revised version of your manuscript taking the referees' suggestions on board, along with a detailed point-by-point response addressing all referees' comments. Please note that it is The EMBO Journal policy to allow only a single round of major revision, and acceptance of your manuscript will therefore depend on the completeness of your responses in this revised version. Given the nature of the raised concerns, it would be useful to discuss your revision plan already during the initial phase of your revision. You are very welcome to share with me a draft point-by-point response letter/revision plan explaining if there are any points you do not agree with or cannot address, and we could then also arrange a video call to discuss further, if necessary.

We generally allow three months as standard revision time (15 April, 2026). As a matter of policy, competing manuscripts published during this period will not negatively impact our assessment of the conceptual advance presented by your study. However, we request that you contact us as soon as possible upon publication of any related work, to discuss how to proceed. Should you foresee a problem in meeting this three-month deadline, please let us know in advance and we will be able to grant an extension.

Please let me know if you have any questions or comments that you would like to discuss with me. Thank you for the opportunity to consider your work for publication in The EMBO Journal. I look forward to your revision.

Best regards,

Ioannis

Ioannis Papaioannou, PhD
Editor, The EMBO Journal
i.papaioannou@embojournal.org

*****
Instructions for preparing your revised manuscript
*****

1. When you are ready to submit the revision, please upload:

- A Word file of the manuscript text (including legends of main Figures, EV Figures and Tables). Please make sure that changes are highlighted (or "tracked") to be clearly visible.

- Individual production-quality figure files (one file per figure). When assembling your figures, please refer to our figure preparation guidelines in order to ensure proper formatting and readability in print as well as on screen:
https://bit.ly/EMBOPressFigurePreparationGuideline
If the data shown in a figure are obtained from n {less than or equal to} 2, please use scatter plots showing the individual data points.
Please also include scale bars in all microscopy images.
The following points must be specified in each figure legend:
i. the name of the statistical test used to generate error bars and P values

ii. the number (n) of independent experiments (please specify technical or biological replicates) underlying each data point (discussion of statistical methodology can be reported in the Materials and Methods section, but figure legends should contain a basic description of n, P, and the test applied)
iii. the nature of the bars and error bars (s.d., s.e.m.).
See also guidelines for figure legends: https://www.embopress.org/page/journal/14602075/authorguide#figureformat

- A point-by-point response to the referees' comments, with a detailed description of the changes made (as a word file). All referees' concerns must be fully addressed and their suggestions taken on board. When preparing your letter of response to the referees' comments, please bear in mind that this will form part of the Review Process File and will therefore be available online to the community. Please note that you have the possibility to opt out of the transparent process at any stage prior to publication by letting the editorial office know (contact@embojournal.org); if you do opt out, the Review Process File link will point to the following statement: "No Review Process File is available with this article, as the authors have chosen not to make the review process public in this case.". For more details on our Transparent Editorial Process, please visit our website: https://www.embopress.org/page/journal/14602075/authorguide#transparentprocess

- Expanded View (EV) files (replacing Supplementary Information) that are collapsible/expandable online. A maximum of 5 EV Figures can be typeset. EV Figures should be cited as "Figure EV1, Figure EV2" etc. in the text, and their respective legends should be included in the manuscript file after the legends of regular figures. See detailed instructions regarding Expanded View files here:
https://www.embopress.org/page/journal/14602075/authorguide#expandedview

- For the figures that you do NOT wish to display as Expanded View figures, they should be bundled together with their legends in a single PDF file called "Appendix", which should start with a short Table of Contents (including page numbers). Appendix figures should be referred to in the main text as: "Appendix Figure S1, Appendix Figure S2" etc. Please see detailed instructions here: https://www.embopress.org/page/journal/14602075/authorguide#expandedview

- A complete author checklist, which you can download from our author guidelines (https://www.embopress.org/page/journal/14602075/authorguide). Please note that the checklist will also be part of the Review Process File.

2. Please note that no statistics should be calculated and shown in Figures if n=2. Please also note that each p value should be reported as an exact value.

3. Before submitting your revision, primary datasets (and computer code, where appropriate) produced in this study need to be deposited in appropriate public databases (see https://www.embopress.org/page/journal/14602075/authorguide#dataavailability). In particular, all datasets such as DNA or RNA sequencing data, or mass spectrometry data generated in the study must be deposited in appropriate repositories. The accession numbers, database, and the specific URLs (links) should be listed in a formal "Data availability" section (placed after Methods), following the example below:

"The RNA-seq datasets produced in this study are available in the following database:
Gene Expression Omnibus GSE46843 (https://www.ncbi.nlm.nih.gov/geo/query/acc.cgi?acc=GSE46843)"

*** All links should resolve to a page where the data can be accessed. ***

*** Please remember to provide in the Data availability section of your revised manuscript reviewer passwords if the datasets are not yet public. ***

*** The Data Availability Section is restricted to new primary data that are part of this study. In case you have no data that require deposition in a public database, please state so instead of referring to the database: "Our study includes no data deposited in public repositories." under the heading "Data availability". ***

4. The materials and methods need to be described in the manuscript using our structured methods format, which is now required for all research articles. According to this format, the Methods section includes a single "Reagents and Tools Table" - listing key reagents, experimental models, software and relevant equipment including their sources and relevant identifiers- followed by a "Methods and Protocols" section describing the methods. Please download and fill our Reagents and Tools Table template (.docx), which you can find in our author guide:
https://www.embopress.org/page/journal/14602075/authorguide#structuredmethods. When submitting your revised manuscript, please do not include the Reagents and Tools Table in the Methods section of the manuscript but instead upload it as a separate file choosing the file type "Reagent Table".

5. Please check that the title and the abstract of the manuscript are brief, yet explicit, even to non-specialists. The length of the title should not exceed 100 characters, and the abstract should be a single paragraph not exceeding 175 words.

6. Please also note our reference format: https://www.embopress.org/page/journal/14602075/authorguide#referencesformat.

7. At EMBO Press we ask authors to provide source data for the main manuscript figures. You will receive a separate email with instructions for providing source data with your revised manuscript, including how to upload and organize the files.

8. Please remember: digital image enhancement is acceptable practice, as long as it accurately represents the original data and conforms to community standards. If a figure has been subjected to significant electronic manipulation, this must be noted in the figure legend or in the "Materials and Methods" section. The editors reserve the right to request original versions of figures and the original images that were used to assemble the figure.

9. Our journal encourages inclusion of data citations in the reference list to directly cite datasets that were obtained from public databases. Data citations in the article text are distinct from normal bibliographical citations and should directly link to the database records from which the data can be accessed. In the main text, data citations are formatted as follows: "Data ref: Smith et al, 2001" or "Data ref: NCBI Sequence Read Archive PRJNA342805, 2017". In the Reference list, data citations must be labeled with "[DATASET]". A data reference must provide the database name, accession number/identifiers, and a resolvable link to the landing page from which the data can be accessed at the end of the reference. Further instructions are available at: https://www.embopress.org/page/journal/14602075/authorguide#referencesformat.

10. We request authors to consider both actual and perceived competing interests. Please review our policy (https://www.embopress.org/page/journal/14602075/authorguide#conflictsofinterest) and update your competing interests statement if necessary. Please name this section 'Disclosure and competing interests statement' and place it after the Acknowledgements section.

11. Please note that all corresponding authors are required to provide an ORCID ID upon submission of a revised manuscript (https://orcid.org/). Please find instructions on how to link your ORCID ID to your account in our manuscript tracking system in our Author guidelines
(https://www.embopress.org/page/journal/14602075/authorguide#authorshipguidelines).

12. We use CRediT to specify the contributions of each author in the journal submission system. CRediT replaces the author contribution section, which should be removed from the manuscript. Please use the free text box to provide more detailed descriptions. See also guide to authors:
https://www.embopress.org/page/journal/14602075/authorguide#authorshipguidelines.

13. Further information is available in our Guide For Authors: https://www.embopress.org/page/journal/14602075/authorguide

14. We would also welcome the submission of cover suggestions or motifs to be used by our Graphics Illustrator in designing a cover.

15. Please use the link below to submit your revision:
https://emboj.msubmit.net/cgi-bin/main.plex

Referee #1:

Courey-Ghaouzi et al. describe the use of CD24 and CD27 markers to delineate atypical B-cell (ABC) differentiation stages and memory origin, also based on the identification of atypical B cells using CD11c. They find that ABCs originate predominantly from memory B cells, likely shaped by activation outside of germinal centers. Importantly, they link ABC phenotypes and differentiation stages with functionality by analyzing antibody-secreting cell differentiation potential, which is diminished in late-stage ABCs. The authors do a good job introducing ABCs in malaria and other biological and clinical contexts while highlighting gaps in the field and linking their results with the existing literature in the Discussion, reconciling prior data with their results and proposed framework. The use of tonsillar samples to compare with peripheral blood ABCs is a nice addition. Unfortunately, the relationship of the findings and conclusions to malaria is weak.

Some concerns and limitations:

Limitations are not explicitly discussed.

A major limitation is the very small sample size (N = 4 for the CITE-seq analyses). How were these four individuals selected? The main cell clusters are, however, validated by flow cytometry.

Although samples from malaria-infected individuals were used, the findings are largely general and not malaria-specific. The B cells analyzed are not malaria-specific, and it is unclear how they change over time following resolution of infection.

Related to this point, there is only one section referring to longitudinal analyses; however, all analyses appear to have been performed on acute samples rather than longitudinal samples (e.g., 10 days and 1 year post-infection). This is only briefly mentioned with reference to Fig. S3B.

Although participant characteristics are described in Table S1, additional description in the Results section would help readers contextualize the data. Particularly relevant are the origin of the samples, whether participants were previously exposed or not, and age and sex, as all of these factors may affect ABC frequencies and phenotypes. No analyses with the flow data are presented comparing ABCs in differentially exposed individuals or stratifying by sex. While the sample size for single-cell analyses is limited, such analyses could potentially be performed for the flow cytometry data.

Analysis of clonal diversity would have been more informative if performed with consideration of malaria exposure levels.

The authors state that the CD24^{+} intermediate ABC2 subset likely originates from memory B cells. How does this need to be interpreted if this subset is detected only 10 days after acute infection in previously naïve individuals? In healthy individuals without immune perturbation or activation, why would these subsets differentiate?

The authors state in the Discussion: "Notably, ABCs encompassing various maturation stages were detectable under homeostatic conditions and persisted for up to one year following malaria infection, even in the absence of antigenic re-exposure." Where is the evidence showing persistence for up to one year following malaria infection?

Regarding the single-cell transcriptomic analysis Methods section, additional information should be provided. Was doublet filtering considered? Was data scaling performed? How many principal components were retained for clustering, and how was this determined? What minimum log-fold-change and adjusted p-value thresholds were used for differential gene expression analyses?

Minor:
- Review sentence line 125
- Figure S1E: is the statistical analysis done comparing malaria vs Hantavirus? not clear form the figure legend.
- In relation to sentence "Overall, there was more expansion among B cells 127 that expressed >1 ABC marker", this is only clear for FcRL5 and CXCR3
- Figure legend 1 needs grammar and typo revision.
- Review reference to Fig S3C line 225.

Referee #2:

Atypical B cells (ABC) have raised much interest in recent years, as it became evident that these B cells occur at increased frequencies in various chronic infections and autoimmune diseases. There is still quite some confusion about the identity and function of these cells, as different groups used different marker constellations to identify these cells, and as there is quite some heterogeneity in distinct disease settings or healthy individuals. The authors of the present manuscript focused on malaria patients (with some analyses also for blood from healthy donors or tonsils from patients undergoing routine tonsillectomy) and applied a broad approach with all relevant ABC markers, studying both single cell transcriptomes, and surface marker expression by flow cytometry. Based on their analyses, they defined subsets of ABC (early, intermediate 1 and 2, late) and propose a trajectory for the development of these subsets. The detailed analysis of the cells goes beyond most of the prior studies on ABC and is hence a valuable source of data about ABCs. However, there is a main problem with the differentiation scenario that is presented as the main result of the work, as is specified below.

Main criticism

A main conclusion of the study is the distinction of three (or four) subsets of ABC, early, intermediate (1 and 2) and late. This differentiation path is mainly based on trajectories interpreted from single cell RNA sequencing pattern and the differential expression of selected key differentiation markers. However, the concept that this is the pathway of their differentiation is not easily brought together with other key features of these subsets. First, early ABC are enriched for class-switched B cells, whereas intermediate and late ABC are mostly IgM- (and/or IgD-) expressing (lines 187-188, and lines 325-332). However, a class-switched B cell can never revert into an IgM-expressing B cells, meaning that none of the class-switched early ABC can ever become a non-class-switched intermediate or late ABC. Second, also the somatic mutation frequencies of the rearranged IgV genes of the subsets appears at variance with the proposed model: Early ABC have a high average mutation frequency (4.5%), intermediate ABC2 are mostly unmutated (0.4), intermediate ABC1 have the highest mutation load (5.4%), and late ABC a quite low average mutation frequency of only 1.6% (lines 334-338). But a highly mutated early ABC or int-ABC1 can never

differentiate into a lowly mutated late ABC. One may speculate that among early ABC only those differentiate into late ABC that have little somatic mutations, or that mostly only non-class-switched early ABC differentiate into late ABC, as the latter are mostly IgM- and/or IgD-positive, but these are only assumptions, and the proposed trajectory from early through intermediate to late does not convincingly fit to the class-switching and somatic hypermutation patterns. These are in my opinion actually stronger markers of differentiation trajectories (because they are genetic and irreversible), than in silico trajectory interpretations from RNA-sequencing data.
It is also confusing that for the two separate trajectories defined in Fig. 2F, one ends in early ABC and the other in late ABC, with no path from early to late ABC. This is also distinct from how the trajectories are being displayed in the graphical abstract, where the two paths converge on late ABC.
Thus, the scenario presented as the main outcome of the work needs to be revisited and either edited or convincing arguments are presented how the class-switching and somatic mutation data fit into the concept.

Minor points
a) The term "non-switched memory B cells" is used inconsistently. In the first part of the Results section, IgD+CD27+ B cells are defined as non-switched memory B cells. However, towards the end (p. 21), CD27+ IgD-IgM+ B cells are defined as non-switched memory B cells, whereas now the IgD+IgM+CD27+ B cells are defined as marginal-zone-like non-switched B cells. Indeed, the term marginal-zone like B cells is not defined for the non-expert reader, and it should also be mentioned that there is an ongoing discussion about the identity of these cells, also with strong molecular data that the IgM+IgD+CD27+ B cells are mostly bona fide post-GC memory B cells.

b) Suppl. Table 2: The first row does not show the antibodies, but the antigens. Either add always "anti-" in front of the names of the targets, or specify in the heading "Antibody specific for". The last three in the table are neither antigens or antibodies.

Referee #3:

Since the original description of CD21lo B cells by laus Warnatz in 2002 as a likely distinct subset of human B cells associated with specific pathologies - in this initial instance CBID with non-infectious complications - these cells have been found to be expanded in many other settings of human diseases of immune dysregulation such as chronic pathogen infection (HIV, malaria), autoimmunity (SLE, RA...), severe COVID19 but also found to emerge rapidly in the setting of influenza booster or SARS CoV2 mRNA vaccination. Thus, ascribing specifc functions to CD21lo B cells in settings of health (vaccination) and disease (autoimmunity/immune deficiency) continues to be challenge. This is further heightened by anecdotal (and limited) evidence from humans who have single gene defects that disrupt various aspects of innate and adaptive immunity eg one individual identified to have Tbet deficiency did not exhibit major defects in immunity against infections other than mycobacteria, while ppl deficient in IFNgR also do not exhibit signs of immune dysregulation that might be attributable to a reduction in these B cells.

The complexity of this B cell subset is also exemplified by the highly variable nomenclatures that have been ascribed to these cells, along with suppositions made regarding origin, generation, precursor/progeny relationships and maintenance. Eg CD21lo B cells have been termed (inappropriately) Age associated B cells, atypical memory B cells, activated naïve B cells, double neg B cells etc. these descriptors are incredibly unhelpful when trying to glean ontogenic and functional features.

Despite much confusion and uncertainty, there are certainly features that generally unify the different "types" of CD21lo B cells that have been described. These include phenotypes (eg expression of CD11c, FCRL4/5, CXCR3), transcription factors (expression of TBX21/Tbet, ZEB2) and function (Ag presentation, but diminished Ig production). With these knowledge knowns and unknowns, Courey-Ghaouzi et al have adopted a relatively inclusive flow cytometric based panel to attempt to delineate various subsets and stages of "atypical memory B cells" evolution/differentiation and provide an improved framework to enable greater refinement to our current understanding of such B cells and integrate findings from the general literature into this framework to minimize/obviate various aspects of inconsistency and confusion that currently exist.

Overall, this study has been well thought out/conceived, and very carefully performed/executed with the data generally being presented in clear and logical manner. The data delivers what was hoped for - greater resolution around the transcriptomic, molecular and cellular architecture of "atypical memory B cell subsets", interrelationships between these myriad subsets, and functionality of these subsets and how they may (or may not) differ with respect to state of differentiation. This paper certainly warrants publication, and hopefully my enthusiasm for this work is clear - but it is not without caveats and some shortcomings that need further investigation and/or investigation.

Major comments
1. A major conclusion from this work is the presence of myriad populations of atypical memory B cells in humans, these cells subsets can be detected in circulation and tissues (at least tonsils) in healthy donors, and are differentially distributed in healthy donors vs malaria infection, that they may arise from different precursors and transit thru different towards acquiring effector functions, and that effector function alters along these trajectories ie identification of early, intermed and late "ABCs" was associated with declining capacity to produce Ab in vitro. while this is consistent with other studies and/or conclusions/proposals (even going back to studies of HIV by Moir and Fauci), these data are all derived from comparison to other subsets of atypical

memory B cells. ie reduced Ab secretion by later ABC compared to early ABC. But this really needs to be done in comparison to naïve and/or classical memory B cells (and possibly even GC B cells, which are invoked throughout the paper). Ie include in Fig 3I/Supp Fig 4 - what % of wells seeded with naïve/GC/memory B cells produced Ig etc This will establish the degree of loss of Ab secretion functionality relative to a cell type that probably / possibly gave rise to the ABC subset being tested.
2. Ig secretion in response to an array of in vitro stimulation should also be tested, given findings attributing these ABC type B cells have a dependence on TLR7 (ie papers by I Sanz lab) ie stim with CpG (TLR9) or R848 (TLR7) {plus minus} BCR engagement to assess Ig production.
3. If ABC's lose Ig secretion capacity with progressive differentiation, what is the main function of these later subsets? One very real possibility, as mentioned here but also previously considered Warnatz er al (PMID: 33193306) is Ag presentation. It would be incredibly valuable to assess and compare the ability of the different stages of ABC differentiation (early, intermed, later) to present Ag to T cells/induce T cell activation and/or proliferation to determine if - although Ig secretion capcity declines - these cells acquire concomitant increased capacity to induce CD4 T cell activation.
4. Likewise, levels of SHM need to be compared to classic MBC and GC B cells
5. CXCR3 expression increases then declines along the model of early > intermed > late ABC differentiation, even tho expression of Tbet is maintained or increased along this continuum. This is interesting b/c CXCR3 is a direct target of Tbet (as shown in many systems, but also the inability of human B cells from a Tbet-deficient patient to upregulate CXCR3 expression; PMID: 35867801). Is there an explanation for this loss of CXCR3 expression in late ABC's that express the highest levels of Tbet? See Fig2C, 3C.
6. Line 203: authors proposed to "confirm the clusters' identities by flow cytometry" - CD11c, FCRL5, CXCR3 were assessed. Given the number of DEG that are detectable relatively easily by flow cytometry, I would not consider assessing expression of 3 surface receptors is adequate validation.
7. For Fig 5, data would be more compelling is co-expression of these induced surface markers are included - not just

Other comments
1. Fig 1 legend - E does not show "expansion"; it shows relative frequency of the indicated B cell subsets
2. Fig 2 is titled "Longitudinal CITE Seq..." - but the legend states "cell clusters done on 2601 cells from 12 pooled samples (4 donors and 3 time-points after malaria". If samples are pooled, there is no longer a "longitudinal" aspect to the data
3. Line 248: "10 days after treatment initiation" - pls define meaning of "treatment" in the text
4. Line 374: "In contrast, naïve B cells showed no significant change, likely reflecting their already low baseline CD24 levels..." Naïve B cells have substantial expression of CD24 in a basal/resting state. I think this comment is not strictly accurate. See PMID: 14962195; PMID: 16424179
5. Supp Fig 7 - does "DAF" on x axis refer to CD55 ie decay accelerating factor? If so, how come ie why use this now. If not, pls define what parameter is on the x axis.
6. Numbering of some figures is incorrect eg line194 Supp. Fig 3D, should be C; line 295 Supp Fig 4G should be H?
7. Fig 5E - do the colors refer to IgM/D expression?
8. Line 435: "Almost all CD11c+ B cells were present in the mature B cell compartment" - define mature; presumably these are a mix and transitional/naïve/mem B cells excluded GC Bcells?
9. Many papers need to be cited, esp in the setting of immunity and CD21lo B cells post vaccination
a. Lau et al (PMID: 28783670)
b. Steuten et al PMID: 36858158
c. Bergman et al PMID: 35290571

Referee #1:

Courey-Ghaouzi et al. describe the use of CD24 and CD27 markers to delineate atypical B-cell (ABC) differentiation stages and memory origin, also based on the identification of atypical B cells using CD11c. They find that ABCs originate predominantly from memory B cells, likely shaped by activation outside of germinal centers. Importantly, they link ABC phenotypes and differentiation stages with functionality by analyzing antibody-secreting cell differentiation potential, which is diminished in late-stage ABCs. The authors do a good job introducing ABCs in malaria and other biological and clinical contexts while highlighting gaps in the field and linking their results with the existing literature in the Discussion, reconciling prior data with their results and proposed framework. The use of tonsillar samples to compare with peripheral blood ABCs is a nice addition. Unfortunately, the relationship of the findings and conclusions to malaria is weak.

We thank the reviewer for the positive assessment of the study's scope, our integration of tonsillar and peripheral-blood data, and our linking of ABC phenotype to function. On the relationship to malaria, we would note that this work was not intended as a study of a malaria-specific immune response. We use malaria as a well-characterized model of acute, resolving inflammation in which to define ABC biology. Our conclusions are accordingly directed at the ABC compartment rather than at malaria antigen-specific responses. We have stated this scope explicitly and addressed the temporal behavior directly in the revised manuscript (see Comments 2 and 3).

Some concerns and limitations:

Limitations are not explicitly discussed.

1: A major limitation is the very small sample size (N = 4 for the CITE-seq analyses). How were these four individuals selected? The main cell clusters are, however, validated by flow cytometry.

We agree that the limited CITE-seq cohort (N = 4) is an important consideration. The cohort size is stated explicitly, and the four donors were selected according to predefined criteria, sample availability and viability, comparable age, and a balanced design of two primary-infected (malaria-naïve) and two previously exposed individuals to enable high-quality paired transcriptome and antibody-derived-tag data. As the reviewer notes, the major cell states and overall cluster structure are independently reproduced by flow cytometry in substantially larger cohorts (18 donors with acute malaria, Fig 2, 51 malaria donors with longitudinal analysis, Fig S4; 9 malaria patients and 6 healthy donors, Fig 3; paired blood and tonsil, Fig 6), which we now emphasize, and we clarify throughout where conclusions rest on the N = 4 transcriptomic data versus the broader flow-cytometry validation.

2: Although samples from malaria-infected individuals were used, the findings are largely general and not malaria-specific. The B cells analyzed are not malaria-specific, and it is unclear how they change over time following resolution of infection.

We agree and have adjusted the framing. Our aim is to define ABC biology and heterogeneity using malaria as a well-characterized in vivo model of acute, resolving inflammation, not to claim malaria antigen specificity. The revised abstract and Discussion state this explicitly, and the Discussion now notes that we did not enrich for, or directly identify, antigen-specific B cells, so our conclusions pertain to the broader responding B-cell compartment. The temporal behaviour following resolution is now addressed directly (see Comment 3).

3: Related to this point, there is only one section referring to longitudinal analyses; however, all analyses appear to have been performed on acute samples rather than longitudinal samples (e.g., 10 days and 1 year post-infection). This is only briefly mentioned with reference to Fig. S3B.

We have substantially expanded the longitudinal analysis. A new Results section and Suppl Fig 4 present subset-frequency changes across all sampled time points (acute, day 10, ~1 year): CD11c+ switched and non-switched memory and CD27–CD21– populations peak around day 10 and contract thereafter, and the corresponding time-resolved changes are shown for the CITE-seq clusters (Suppl Fig 4C). These analyses are now explicitly cross-referenced in the Results so that acute and longitudinal observations are clearly distinguished.

4: Although participant characteristics are described in Table S1, additional description in the Results section would help readers contextualize the data. Particularly relevant are the origin of the samples, whether participants were previously exposed or not, and age and sex, as all of these factors may affect ABC frequencies and phenotypes. No analyses with the flow data are presented comparing ABCs in differentially exposed individuals or stratifying by sex. While the sample size for single-cell analyses is limited, such analyses could potentially be performed for the flow cytometry data.

We have added contextual description of the cohort (sample origin and prior-exposure status, malaria-naïve versus previously exposed), and we now stratify ABC frequencies and longitudinal dynamics by prior malaria exposure (Suppl Fig 4): for example, CD11c+ switched memory and CD27–CD21– cells expand more in previously exposed individuals, whereas CD11c+ non-switched memory expansion is independent of prior exposure. Stratification by sex and by graded exposure level falls outside the central aims of the present study, which is focused on defining ABC biology with prior malaria exposure as the main contextual variable. The cohort was not designed or powered to resolve these factors, and we judged that such sub-analyses were unlikely to add interpretable insight bearing on the questions addressed here. We nonetheless regard them as a worthwhile direction for dedicated future studies. Since the manuscript has also become quite extensive, we have prioritized additional text describing new results and analyses and refer to Table S1 for clinical details associated with each figure.

5: Analysis of clonal diversity would have been more informative if performed with consideration of malaria exposure levels.

We agree this would be informative. However, our cohort comprises travellers in whom prior exposure is based on self-report and on having grown up in an endemic area,

without a reliable individual-level measure of cumulative exposure. We are therefore unable to stratify clonal diversity by exposure intensity with confidence and have refrained from exposure-dependent conclusions. Where the grouping is robust, we do now provide prior-exposure–stratified frequency analyses (Suppl Fig 4). However, the donor included in the Figure 3 and 4 analyses is considered previously exposed and it therefore makes sense that the person has previous GC-derived memory that could drive an Early ABC -> Int-ABC1 differentiation.

6: The authors state that the CD24$^+$ intermediate ABC2 subset likely originates from memory B cells. How does this need to be interpreted if this subset is detected only 10 days after acute infection in previously naïve individuals? In healthy individuals without immune perturbation or activation, why would these subsets differentiate?

We understand that this requires clarification, and we will revise the manuscript to state this more explicitly. By "likely originates from memory B cells," we refer to originating from the broader pre-existing antigen-experienced, non-switched memory-like compartment (i.e., CD27$^+$IgD$^+$ marginal zone-like cells), rather than necessarily malaria-specific GC-derived memory. Thus, "previously naïve" in our cohort should be interpreted as malaria-naïve rather than immunologically naïve.

The detection of CD24$^+$ intermediate ABC2 cells by day 10 is consistent with rapid recruitment and phenotypic remodelling of antigen-experienced B cells under strong inflammatory/innate cues during acute infection and is compatible with extrafollicular activation pathways that have been proposed to generate ABC-like states on faster timescales than de novo germinal center-derived differentiation.

Regarding their presence in healthy individuals, we do not interpret "healthy" as absence of immune stimulation. Clinically healthy donors can have ongoing or recent immune stimulation (e.g., subclinical infections, mucosal antigen exposure, vaccination history) that transiently populate activated/intermediate B-cell states without overt disease. Consistent with this model, our Figure 1 shows that a substantial fraction of proliferating B cells express ABC-associated markers, supporting the view that acquisition of ABC features can be part of normal activation dynamics and helping to explain the early appearance and baseline presence of intermediate ABC states. We and other show that they greatly expand during strong inflammatory stimuli though. In addition, ABC frequencies have been reported to increase with age in the literature, consistent with cumulative antigen exposure and immune stimulation over time and higher baseline inflammation contributing to their baseline detectability in healthy individuals.

7: The authors state in the Discussion: "Notably, ABCs encompassing various maturation stages were detectable under homeostatic conditions and persisted for up to one year following malaria infection, even in the absence of antigenic re-exposure." Where is the evidence showing persistence for up to one year following malaria infection?

We agree that this statement was not sufficiently supported in the previous version of the manuscript. The evidence comes from our longitudinal sampling, where ABC subsets remain detectable at the ~1-year post-infection time point in the same individuals, in the

absence of documented reinfection during follow-up. This data was included in a prior version but had unfortunately been removed in the previous submission.

We have added the supporting evidence. The longitudinal data now show ABC subsets remaining detectable at ~1 year (Suppl Fig 4), and we have added this citation to the relevant Discussion sentence and tempered the claim by noting that unmeasured antigenic exposures beyond documented reinfection cannot be fully excluded.

8: Regarding the single-cell transcriptomic analysis Methods section, additional information should be provided. Was doublet filtering considered? Was data scaling performed? How many principal components were retained for clustering, and how was this determined? What minimum log-fold-change and adjusted p-value thresholds were used for differential gene expression analyses?

We have expanded the Methods accordingly. We now specify data scaling (ScaleData); the number of principal components retained and how this was determined (ElbowPlot for the targeted CITE-seq data and JackStraw corroborated by ElbowPlot for the 10X data; 10/15 and 20 PCs, respectively); and the differential-expression thresholds (Wilcoxon rank-sum; min.pct = 0.25; log2FC ≥ 0.25; significance at adjusted $p \leq 0.05$ and $|log2FC| > 1$). Regarding doublets, formal computational doublet detection was not separately re-applied: the targeted CITE-seq B cells derive from a parent dataset that had already passed quality control, and for the 10X data we used an upper detected-gene threshold (<3,500) as a conservative doublet proxy; in an enriched, low-heterogeneity B-cell population, residual homotypic doublets are not expected to generate artifactual clusters. This is now stated in the Methods, and the full pipeline remains available in the shared code repository.

Minor:

- Review sentence line 125

The sentence has been revised for clarity.

- Figure S1E: is the statistical analysis done comparing malaria vs Hantavirus? not clear form the figure legend.

The legend now states that the comparison is between malaria and hantavirus.

- In relation to sentence "Overall, there was more expansion among B cells 127 that expressed >1 ABC marker", this is only clear for FcRL5 and CXCR3

We may not have described this analysis clearly enough, and we would like to clarify. Figure 1E reports the expansion (log2 fold-change, Ki67+ vs Ki67–) of each Boolean marker combination of the four assessed markers, with statistics calculated for every combination. Across these, all but one combination were significantly expanded among proliferating (Ki67+) B cells, and combinations co-expressing more than one ABC marker were predominantly expanded, which is the basis for the statement. We would also note that combinations containing FcRL5 and/or CXCR3 are themselves >1-marker populations and are therefore consistent with, rather than exceptions to, the pattern described. The statement is thus supported by the per-combination analysis already shown in Fig 1E, and we are happy to adjust the wording of the sentence and the Fig 1E legend to make the per-combination basis explicit and avoid any ambiguity.

- Figure legend 1 needs grammar and typo revision.
The legend has been corrected (including "ANOVA" and the statistics wording).

- Review reference to Fig S3C line 225.
The cross-reference has been checked and corrected.

Referee #2:

Atypical B cells (ABC) have raised much interest in recent years, as it became evident that these B cells occur at increased frequencies in various chronic infections and autoimmune diseases. There is still quite some confusion about the identity and function of these cells, as different groups used different marker constellations to identify these cells, and as there is quite some heterogeneity in distinct disease settings or healthy individuals. The authors of the present manuscript focused on malaria patients (with some analyses also for blood from healthy donors or tonsils from patients undergoing routine tonsillectomy) and applied a broad approach with all relevant ABC markers, studying both single cell transcriptomes, and surface marker expression by flow cytometry. Based on their analyses, they defined subsets of ABC (early, intermediate 1 and 2, late) and propose a trajectory for the development of these subsets. The detailed analysis of the cells goes beyond most of the prior studies on ABC and is hence a valuable source of data about ABCs. However, there is a main problem with the differentiation scenario that is presented as the main result of the work, as is specified below.

We thank the reviewer for recognizing the depth of the analysis and for a central methodological challenge to our differentiation model. A misunderstanding appears to have arisen around our branching interpretation, and we welcome the opportunity to set it out explicitly and to ground it more firmly in the genetic (V(D)J) data.

Main criticism

1: A main conclusion of the study is the distinction of three (or four) subsets of ABC, early, intermediate (1 and 2) and late. This differentiation path is mainly based on trajectories interpreted from single cell RNA sequencing pattern and the differential expression of selected key differentiation markers. However, the concept that this is the pathway of their differentiation is not easily brought together with other key features of these subsets. First, early ABC are enriched for class-switched B cells, whereas intermediate and late ABC are mostly IgM- (and/or IgD-) expressing (lines 187-188, and lines 325-332). However, a class-switched B cell can never revert into an IgM-expressing B cells, meaning that none of the class-switched early ABC can ever become a non-class-switched intermediate or late ABC.

We fully agree that class switching is irreversible, and we did not intend to imply that class-switched Early ABC are a common precursor giving rise to non-switched intermediate or late states. We have revised the text and figure legends to make the branching interpretation explicit: Early ABC align with the mutated, frequently class-switched Int-ABC1 axis, whereas

Int-ABC2 is a distinct, predominantly non-switched population with low SHM that does not derive from Early ABC. Distinct origins thus converge on a shared late phenotype, with no implied reversion from switched to IgM/IgD-expressing states, and we have removed any wording suggesting an obligatory single precursor.

2: Second, also the somatic mutation frequencies of the rearranged IgV genes of the subsets appears at variance with the proposed model: Early ABC have a high average mutation frequency (4.5%), intermediate ABC2 are mostly unmutated (0.4), intermediate ABC1 have the highest mutation load (5.4%), and late ABC a quite low average mutation frequency of only 1.6% (lines 334-338). But a highly mutated early ABC or int-ABC1 can never differentiate into a lowly mutated late ABC. One may speculate that among early ABC only those differentiate into late ABC that have little somatic mutations, or that mostly only non-class-switched early ABC differentiate into late ABC, as the latter are mostly IgM- and/or IgD-positive, but these are only assumptions, and the proposed trajectory from early through intermediate to late does not convincingly fit to the class-switching and somatic hypermutation patterns. These are in my opinion actually stronger markers of differentiation trajectories (because they are genetic and irreversible), than in silico trajectory interpretations from RNA-sequencing data.

We agree that SHM provides a stringent, genetically irreversible constraint, and we have aligned our interpretation with it. Our clonal-lineage analysis detected relatively few lineages (consistent with CD11c-based rather than antigen-specific sorting), and among these, several bridged Early ABC and the highly mutated Int-ABC1 (Fig 4D–E). The distinct origin of Int-ABC2 is supported primarily by its predominantly unmutated, IGHM-enriched BCR repertoire rather than by demonstrated clonal relationships; we therefore interpret Int-ABC2 as entering from a distinct, antigen-experienced but non-switched, low-SHM precursor pool, and the intermediate SHM of Late ABC as reflecting contributions from both intermediate axes rather than derivation from highly mutated precursors alone. We thus frame SHM and isotype as evidence for multiple entry points rather than a single serial trajectory. We acknowledge in the Discussion that we did not benchmark SHM against classical memory and GC B cells; targeted bulk IgH sequencing of sorted compartments would address this directly but was not feasible with the limited remaining patient material, and we flag it as an important next step.

3: It is also confusing that for the two separate trajectories defined in Fig. 2F, one ends in early ABC and the other in late ABC, with no path from early to late ABC. This is also distinct from how the trajectories are being displayed in the graphical abstract, where the two paths converge on late ABC.
Thus, the scenario presented as the main outcome of the work needs to be revisited and either edited or convincing arguments are presented how the class-switching and somatic mutation data fit into the concept.

We have clarified this. The two separate endpoints in Fig 2F reflect a known property of trajectory inference: Slingshot relies on local neighborhood structure in the low-dimensional embedding and cannot reliably connect non-adjacent clusters; because the CD11c+ populations sit apart from the late-ABC state in the embedding, it recovers two local trajectories rather than a single continuous path. We now state this explicitly in the Results. Our inference of convergence does not rest on pseudotime but on the in vitro differentiation

data (Fig 5), in which activated B cells of different origins converge on a common double-negative phenotype. This distinction, between the locally inferred trajectory structure and the experimentally supported convergence, is now made explicit in the Results, and we are happy to add a corresponding note to the Fig 2F legend and the graphical abstract.

Minor points

a) The term "non-switched memory B cells" is used inconsistently. In the first part of the Results section, IgD+CD27+ B cells are defined as non-switched memory B cells. However, towards the end (p. 21), CD27+ IgD-IgM+ B cells are defined as non-switched memory B cells, whereas now the IgD+IgM+CD27+ B cells are defined as marginal-zone-like non-switched B cells. Indeed, the term marginal-zone like B cells is not defined for the non-expert reader, and it should also be mentioned that there is an ongoing discussion about the identity of these cells, also with strong molecular data that the IgM+IgD+CD27+ B cells are mostly bona fide post-GC memory B cells.

We have standardized the terminology, now define “marginal-zone-like” (CD27+IgD+IgM+) at first use, and added a note acknowledging the ongoing debate, including evidence that many IgM+IgD+CD27+ cells represent bona fide post-germinal-center memory.

b) Suppl. Table 2: The first row does not show the antibodies, but the antigens. Either add always "anti-" in front of the names of the targets, or specify in the heading "Antibody specific for". The last three in the table are neither antigens or antibodies.

Supplementary Table 2 has been corrected: the target column is now headed appropriately (“Antibody specific for”), and the non-antibody reagents (e.g., viability dye) are labeled as such.

Referee #3:

Since the original description of CD21lo B cells by laus Warnatz in 2002 as a likely distinct subset of human B cells associated with specific pathologies - in this initial instance CBID with non-infectious complications - these cells have been found to be expanded in many other settings of human diseases of immune dysregulation such as chronic pathogen infection (HIV, malaria), autoimmunity (SLE, RA...), severe COVID19 but also found to emerge rapidly in the setting of influenza booster or SARS CoV2 mRNA vaccination. Thus, ascribing specifc functions to CD21lo B cells in settings of health (vaccination) and disease (autoimmunity/immune deficiency) continues to be challenge. This is further heightened by anecdotal (and limited) evidence from humans who have single gene defects that disrupt various aspects of innate and adaptive immunity eg one individual identified to have Tbet deficiency did not exhibit major defects in immunity against infections other than mycobacteria, while ppl deficient in IFNgR also do not exhibit signs of immune dysregulation that might be attributable to a reduction in these B cells.

The complexity of this B cell subset is also exemplified by the highly variable nomenclatures that have been ascribed to these cells, along with suppositions made regarding origin,

generation, precursor/progeny relationships and maintenance. Eg CD21lo B cells have been termed (inappropriately) Age associated B cells, atypical memory B cells, activated naïve B cells, double neg B cells etc. these descriptors are incredibly unhelpful when trying to glean ontogenic and functional features.

Despite much confusion and uncertainty, there are certainly features that generally unify the different "types" of CD21lo B cells that have been described. These include phenotypes (eg expression of CD11c, FCRL4/5, CXCR3), transcription factors (expression of TBX21/Tbet, ZEB2) and function (Ag presentation, but diminished Ig production). With these knowledge knowns and unknowns, Courey-Ghaouzi et al have adopted a relatively inclusive flow cytometric based panel to attempt to delineate various subsets and stages of "atypical memory B cells" evolution/differentiation and provide an improved framework to enable greater refinement to our current understanding of such B cells and integrate findings from the general literature into this framework to minimize/obviate various aspects of inconsistency and confusion that currently exist.

Overall, this study has been well thought out/conceived, and very carefully performed/executed with the data generally being presented in clear and logical manner. The data delivers what was hoped for - greater resolution around the transcriptomic, molecular and cellular architecture of "atypical memory B cell subsets", interrelationships between these myriad subsets, and functionality of these subsets and how they may (or may not) differ with respect to state of differentiation. This paper certainly warrants publication, and hopefully my enthusiasm for this work is clear - but it is not without caveats and some shortcomings that need further investigation and/or investigation.

We are grateful for the reviewer's enthusiasm and for comments that directly motivated the new functional characterization in Fig 7. We share the reviewer's view that inconsistent nomenclature for CD21lo B cells (age-associated, atypical memory, activated naïve, double-negative) has obscured their ontogeny and function, and that the unifying features noted (CD11c, FCRL4/5, CXCR3; TBX21/ZEB2; antigen presentation with diminished immunoglobulin production) are the axes a better framework must resolve. Our inclusive panel was intended to place these descriptors onto a common differentiation framework rather than treat them as discrete entities. The reviewer's major comments prompted us to test that framework's functional predictions directly in Fig 7, and we address each point in turn below.

Major comments

1. A major conclusion from this work is the presence of myriad populations of atypical memory B cells in humans, these cells subsets can be detected in circulation and tissues (at least tonsils) in healthy donors, and are differentially distributed in healthy donors vs malaria infection, that they may arise from different precursors and transit thru different towards acquiring effector functions, and that effector function alters along these trajectories ie identification of early, intermed and late "ABCs" was associated with declining capacity to produce Ab in vitro. while this is consistent with other studies and/or conclusions/proposals (even going back to studies of HIV by Moir and Fauci), these data are all derived from comparison to other subsets of atypical memory B cells. ie reduced Ab secretion by later ABC compared to early ABC. But this really needs to be done in comparison to naïve and/or

classical memory B cells (and possibly even GC B cells, which are invoked throughout the paper). Ie include in Fig 3I/Supp Fig 4 - what % of wells seeded with naïve/GC/memory B cells produced Ig etc This will establish the degree of loss of Ab secretion functionality relative to a cell type that probably / possibly gave rise to the ABC subset being tested.

We agree that benchmarking against canonical compartments would add context, and we have framed our functional conclusions accordingly as relative differences within the ABC continuum (Fig 7D–E). We did not implement naïve, classical-memory and GC controls within the same single-cell-density culture: the assay requires sorting multiple subsets at single-cell density with high viability and sufficient yield from each donor, and because all populations must derive from each donor to control for inter-donor and inter-experimental variation, adding further populations would have reduced cell numbers and data quality and effectively required regenerating the dataset. We therefore frame our functional conclusions explicitly as relative differences across ABC stages rather than as absolute comparisons to naïve or memory compartments.

2. Ig secretion in response to an array of in vitro stimulation should also be tested, given findings attributing these ABC type B cells have a dependence on TLR7 (ie papers by I Sanz lab) ie stim with CpG (TLR9) or R848 (TLR7) {plus minus} BCR engagement to assess Ig production.

We agree that responsiveness across a broader stimulation space is informative. Because our single-B-cell antibody assay depends on CD40L-expressing feeder cells for plasma-cell survival, which constrains systematic variation of TLR stimuli in that format, we addressed the TLR-dependence question with a complementary readout: intracellular cytokine production by ABC subsets after anti-Ig/CD40L, CpG (TLR9), R848 (TLR7/8) and PAM2CSK4 (TLR2) stimulation (Fig 7G–J). This shows, for example, that BCR/CD40 co-stimulation drives TNFα most strongly in CD27-expressing subsets while CpG broadens responsiveness across subsets. A systematic dissection of TLR ± BCR effects specifically on antibody secretion across subsets remains an important goal for dedicated future work.

3. If ABC's lose Ig secretion capacity with progressive differentiation, what is the main function of these later subsets? One very real possibility, as mentioned here but also previously considered Warnatz er al (PMID: 33193306) is Ag presentation. It would be incredibly valuable to assess and compare the ability of the different stages of ABC differentiation (early, intermed, later) to present Ag to T cells/induce T cell activation and/or proliferation to determine if - although Ig secretion capcity declines - these cells acquire concomitant increased capacity to induce CD4 T cell activation.

We agree this is a central question and address it directly. Using the 10X dataset, pathway (IPA) and per-cell module-score (UCell) analyses show a progressive functional shift along the axis: early subsets are enriched for cytokine-production and antibody-secretion programs, whereas late subsets are enriched for phagocytosis and antigen-presentation programs (Fig 7A–C). We complemented this with a microbial-particle (E. coli) association assay, in which association is highest in Late ABC and lowest in Early ABC (Fig 7F), and with the intracellular cytokine analysis above (Fig 7G–J). Together these indicate that as antibody-secretion potential declines, late ABC acquire scavenger and antigen-presentation features.

We note, however, that the microbial-particle assay measures particle association rather than confirmed internalization; we attempted to distinguish active uptake using cytochalasin D treatment but were unable to establish a reliable assay in this system, and we therefore cannot at present formally confirm phagocytosis. We accordingly describe these as scavenger- and antigen-presentation–associated features and flag this as a limitation. We did not include a direct autologous T-cell activation/proliferation assay, which would require substantial additional viable sorted cells and matched co-culture systems beyond the available material. We present the transcriptomic and functional data as convergent evidence and note this in the text.

4. Likewise, levels of SHM need to be compared to classic MBC and GC B cells

As in our response to Reviewer 2 (Main point 2), we agree this would strengthen the repertoire interpretation. It requires targeted bulk IgH sequencing of additional sorted compartments and, given the limited remaining material and the technical demands of obtaining sufficient cells, was not feasible within this revision. We therefore note it explicitly in the Discussion and interpret SHM levels only within the analyzed ABC populations.

5. CXCR3 expression increases then declines along the model of early > intermed > late ABC differentiation, even tho expression of Tbet is maintained or increased along this continuum. This is interesting b/c CXCR3 is a direct target of Tbet (as shown in many systems, but also the inability of human B cells from a Tbet-deficient patient to upregulate CXCR3 expression; PMID: 35867801). Is there an explanation for this loss of CXCR3 expression in late ABC's that express the highest levels of Tbet? See Fig2C, 3C.

This is an interesting point, which we address here. We now profile chemokine-receptor and migration markers (CXCR3, CCR1, CXCR5, CD69) across the ABC subsets in blood and tonsil (Fig 6H–J). Regarding the apparent divergence: while T-bet is required to induce CXCR3, it is not necessarily sufficient to maintain its surface expression — CXCR3 undergoes ligand-driven internalization and desensitization in highly activated cells, and CXCR3-high cells may preferentially egress from blood into tissues, leaving a CXCR3-low late compartment in circulation. We offer these as candidate, non-exclusive explanations and do not imply that CXCR3 is governed solely by T-bet levels. As these interpretations remain speculative, we prefer to provide them in this response rather than state them as firm conclusions in the manuscript.

6. Line 203: authors proposed to "confirm the clusters' identities by flow cytometry" - CD11c, FCRL5, CXCR3 were assessed. Given the number of DEG that are detectable relatively easily by flow cytometry, I would not consider assessing expression of 3 surface receptors is adequate validation.

We agree and have broadened the validation considerably. Figure 6 now reports per-subset expression of a larger panel (CCR1, CXCR3, CXCR5, CD69, T-bet, FcRL3, FcRL4, FcRL5, NKG7 and CD86) across the CD24/CD27-defined ABC subsets in paired blood and tonsil. We present this as an orthogonal protein-level mapping of the major transcriptomic states rather than a comprehensive proteomic characterization, as stated in the text.

7. For Fig 5, data would be more compelling is co-expression of these induced surface markers are included - not just

We have added the requested co-expression analysis. Using Boolean gating, we now show co-expression of the induced ABC-associated markers at the single-cell level during in vitro differentiation (Suppl Fig 7D), allowing readers to see that marker induction occurs in overlapping rather than wholly distinct cell fractions, mirroring the pattern observed ex vivo in malaria and hantavirus infection.

Other comments

1. Fig 1 legend - E does not show "expansion"; it shows relative frequency of the indicated B cell subsets

The legend now distinguishes panel D (relative frequency) from panel E (log2 fold-change, i.e., expansion).

2. Fig 2 is titled "Longitudinal CITE Seq..." - but the legend states "cell clusters done on 2601 cells from 12 pooled samples (4 donors and 3 time-points after malaria". If samples are pooled, there is no longer a "longitudinal" aspect to the data

Clarified: panel A pools the time points for clustering, while the longitudinal frequency analysis is presented separately in Suppl Fig 4; the title and legend now make this explicit.

3. Line 248: "10 days after treatment initiation" - pls define meaning of "treatment" in the text

Defined: “treatment” refers to antimalarial treatment with artemether-lumefantrine; the relevant samples were taken 10 days after treatment initiation.

4. Line 374: "In contrast, naïve B cells showed no significant change, likely reflecting their already low baseline CD24 levels..." Naïve B cells have substantial expression of CD24 in a basal/resting state. I think this comment is not strictly accurate. See PMID: 14962195; PMID: 16424179

Corrected: we have removed the “already low baseline CD24” rationale and revised the interpretation accordingly.

5. Supp Fig 7 - does "DAF" on x axis refer to CD55 ie decay accelerating factor? If so, how come ie why use this now. If not, pls define what parameter is on the x axis.

Defined: DAF denotes CD55 (decay-accelerating factor). The axis label and legend now specify this.

6. Numbering of some figures is incorrect eg line194 Supp. Fig 3D, should be C; line 295 Supp Fig 4G should be H?

All supplementary figure call-outs have been re-checked and corrected with the renumbered figures.

7. Fig 5E - do the colors refer to IgM/D expression?

Clarified: the legend now states that the colors denote the IgM/IgD-defined subsets.

8. Line 435: "Almost all CD11c+ B cells were present in the mature B cell compartment" - define mature; presumably these are a mix and transitional/naïve/mem B cells excluded GC Bcells?

Defined: the mature B-cell compartment refers to CD19+ B cells excluding transitional, plasma and germinal-center cells, as shown in Suppl Fig 8.

9. Many papers need to be cited, esp in the setting of immunity and CD21lo B cells post vaccination

a. Lau et al (PMID: 28783670)
b. Steuten et al PMID: 36858158
c. Bergman et al PMID: 35290571

These references have been added in the relevant context of CD21lo B cells and vaccination.

1st Revision - Editorial Decision 28th Jul 2026

Dear Chris,

Thank you for the submission of your revised manuscript (EMBOJ-2025-121231R) to The EMBO Journal for our consideration, and for your patience during re-review. Your manuscript and your point-by-point responses to the previous comments of all three original referees have now been seen by referees #1 and #2, and we have received their new reports, which you can find appended below.

I am pleased to say that, as you will see, both referees recognize that the revised manuscript is considerably improved, and that most of the initially raised referees' concerns have been adequately addressed in the new strengthened version of the manuscript. Referee #2 has no further comments, while referee #1 identifies a few issues with the new data that were added in response to the original comments of referee #3, and which require clarification in a final revision. They also have a few other minor points, which should also be addressed. Please also address all remaining comments in a point-by-point response detailing any changes to the manuscript.

From the editorial side, there are also a few changes and corrections we need you to make in the final version of the manuscript, before we can move forward with the next steps:

- Please make sure that all funding information provided in the Acknowledgements section of the manuscript is also entered in our online system; information regarding the "Marianne and Marcus Wallenberg Foundation" is currently missing in the system.

- Please note that the References format needs to be updated to the style used by EMBO Press. The list should be alphabetical (not numerical), and "et al." must be used after the names of the first 10 co-authors of each citation. DOI identifiers should be provided only for preprints and datasets that have not been published yet. Please check our author guidelines for more information on our reference format: https://link.springer.com/journal/44318/submission-guidelines#cms-Reference-guidelines.

- Please change heading "Code and data availability" to "Data availability".

- We suggest the following text for the Data availability statement: "Due to institutional data protection policies, transcriptomic and figure source data underlying this study could not be made freely available and have been deposited in a controlled-access repository, including a metadata record describing the existing datasets (doi:10.5281/zenodo.20814279). Requests for access should be directed to Christopher Sundling or Karolinska Institutet's Research Data Office. Applicants must provide details of the proposed research project and evidence of appropriate ethical and regulatory approvals. Where access is approved, data will be shared under a Data Transfer Agreement governing the use, storage, and protection of the data. The R code describing the single-cell RNA sequencing analysis is available via GitHub (https://github.com/SundlingLab/ABCdifferentiation)."

- Thank you for providing a temporary access link to your data deposited in Zenodo for review purposes. The link can now be removed from the Data availability statement.

- Please add a brief statement regarding the protected status of source data in the legend of each affected Figure, e.g. "For data protection reasons, source data for this Figure have been deposited to a controlled-access repository (doi:10.5281/zenodo.20814279).".

- Please rename heading "Conflict of interest" to "Disclosure and competing interests statement".

- Please rename heading "Material and method" to "Methods".

- The Author Contributions statement should be removed from the manuscript file. Instead, we use CRediT to specify the contributions of each author in the journal submission system. Please feel free to use the free text box to provide more detailed descriptions during submission. See also our guide to authors for more information: https://link.springer.com/partners/embo-press/editorial-policies#Authorship.

- The Appendix file needs to be provided in PDF format; its title page should contain heading "Appendix for" followed by the manuscript's title (author and affiliation lists are not necessary) and a Table of Contents including page numbers for all listed items; nomenclature should be "Appendix Figure S#" and "Appendix Table S#" throughout the main manuscript and the Appendix PDF file.

- Please note that EMBO press papers are accompanied online by:
A) a short (2 sentences) summary of the findings and their significance,
B) 2-5 short bullet points highlighting the key results, and
C) a synopsis image in .jpg or .png format that is exactly 550 pixels wide and 300 pixels high. Please note that all text in the synopsis image needs to be legible at the final size. This image should be uploaded as a separate file with the aforementioned file type(s) and pixel dimensions.

Please upload this information along with your revised manuscript (the text for A and B should be provided in a separate Word file).

- Our data editors have checked your Figures and their legends, and raised the following queries. Please address all points below completely in your revised manuscript (all changes should be highlighted or "tracked"):
1. Please define the annotated p-values ****/***/**/* as well as provide the exact p-values for the same in the legend of Figures 6C, H, I, J as appropriate.
2. Please provide the exact p-values in the legends of Figures 1C, E; 2C, 3D-F; 4B, 5A, B, C, E, F; 6K, L; 7F-J.
3. Please define the box plots in terms of minima, maxima, centre, bounds of box and whiskers, and percentile in the legends of Figures 1E, 2J, 4C.
4. Please note that information related to "n" is missing in the legends of Figures 1E, 2J, 4B, C.
5. Please note that the measure of center for the error bars needs to be defined in the legend of Figure 5A.

- Manuscript sections need to be named and ordered as follows: Title page - Abstract - Introduction - Results - Discussion - Methods - Data Availability - Acknowledgements - Disclosure and Competing Interests Statement - References - Figure Legends - main Tables (if there are any) - Expanded View Figure Legends (if there are any).

- Please also note that as part of the EMBO Press transparent editorial process, The EMBO Journal publishes online a Peer Review File along with each accepted manuscript. This File will be published in conjunction with your paper and will include the referee reports, your point-by-point responses and all pertinent correspondence relating to the manuscript. Your Author's Checklist will also be published at the end of the Peer Review File. Please let us know in case you want to remove any data or figures from your point-by-point responses before they are published as part of the Peer Review File. Retaining unpublished data in the Peer Review File means that these count as published and that the Peer Review File would need to be referenced in future publications. Please let the editorial office know in case you want to remove any data from this file (contact@embojournal.org).

We look forward to seeing a final version of your manuscript as soon as possible. Please let us know if you have any questions and use this link to submit your revision: https://emboj.msubmit.net/cgi-bin/main.plex.

Best regards,

Ioannis

Ioannis Papaioannou, PhD
Senior Editor, The EMBO Journal - EMBO Reports - Molecular Systems Biology
i.papaioannou@embojournal.org

Referee #1:

Thank you for the clarifications and for addressing my concerns. Overall, I am satisfied with the responses provided to my and the other reviewers' comments, as well as with the changes made to the manuscript, which has been substantially improved and I think will be of interest for researchers in B cell biology and infectious diseases. However, there are still a few points to address:
- Reviewer 3 pointed out the need to compare the ABC subsets with naïve and classical memory B cells, for instance, in the antibody production assays or in the analysis of SHM levels. The authors justify not including these additional subsets by stating that doing so would require more samples and would not alter the relative comparison between the ABC subsets. While I understand the authors' position, this limitation should be clearly acknowledged in the Discussion.

- The additional data in Figure 7, also requested by Reviewer 3, are a valuable addition to the paper, providing functional information on the ABC subsets. Nevertheless, I have some questions regarding these new analyses:

- Figure 7A does not appear to support the statement in the Results that "the Th1 pathway and MHC class II antigen presentation were more prominent in earlier clusters."

- How confident are the authors that the observed differences in pathways are biologically meaningful? Looking at Figure 7C,

the differences in cytokine expression across clusters are not very convincing. In contrast, differences in phagocytosis or antigen presentation across subsets appear more evident.

- Similar frequencies of TNF-expressing cells are observed in the non-stimulated condition compared with the different stimulation conditions. Moreover, for most stimuli, the frequencies appear to decrease, with the possible exception of Ig + CD40L and perhaps CpG for the CD11c+ subset (Figure 7G). Could the limited effect of stimulation be due to the relatively high frequencies observed in the non-stimulated condition for some individuals? Can the authors provide the gating strategy for TNFα- and IL-10-positive cells? For IL-10, stimulation appears to increase the frequency of positive cells only with CpG and R848.

- In Figure 7I, stimulation effects can be observed for some subsets, particularly with Ig + CD40L and CpG. I think an additional plot comparing the non-stimulated condition with each stimulation (rather than comparing frequencies between subsets within each stimulation) would make it clearer when stimulation has an effect.

Additional minor points:
- Please acknowledge as a limitation that sex, a major determinant of immune responses, was not considered in the analyses, and comment on whether sex-related differences might be expected.
- In Figure 2F, the lineage numbers appear to be exchanged compared with the description in the Results.
- Line 236: by "was independent of memory," do the authors mean "was independent of exposure"?
- The subsection title, "High-resolution transcriptomic and V(D)J sequencing of CD11c+ B Cells reveals additional intermediate ABC states with different plasma cell differentiation potential," should be revised, as the V(D)J sequencing results are presented in the following section.

Referee #2:

The points I had raised in my original report have been adequately addressed.

Point-by-point response to the referees and editorial requests

We thank the edtior and the referees for the re-review of our manuscript. The remaining points raised by Referee #1, the editorial requests and the data editor queries are addressed below. Each comment is reproduced in italics, followed by our response in blue. Changes are indicated in the text with reference to line numbers.

Referee #1

1. *Reviewer 3 pointed out the need to compare the ABC subsets with naïve and classical memory B cells, for instance, in the antibody production assays or in the analysis of SHM levels. The authors justify not including these additional subsets by stating that doing so would require more samples and would not alter the relative comparison between the ABC subsets. While I understand the authors' position, this limitation should be clearly acknowledged in the Discussion.*

Response. Agreed. The limitations paragraph of the Discussion (lines 604 to 605) now states explicitly that naïve and classical memory B cells were not included as comparators, and covers both the antibody production assays and the SHM analysis rather than the latter alone. We note that naïve B cells would define the zero point for SHM and act as a negative control in the secretion assays; the informative comparator that is missing is the classical memory compartment, whose exclusion was already disclosed in the Results.

2. *Figure 7A does not appear to support the statement in the Results that "the Th1 pathway and MHC class II antigen presentation were more prominent in earlier clusters."*

Response. Agreed, and the statement has been removed rather than reassigned. At line 430 the clause ", whereas the Th1 pathway and MHC class II antigen presentation were more prominent in earlier clusters" has been deleted; the sentence now ends at "…Intermediate- and Late-ABCs" and no mention of Th1 remains. Figure 7A shows pathway activation z scores for a selected set of pathways (legend line 1241; Methods line 855) with no associated statistical test, so it cannot support a directional claim; the statement had in fact been derived from the module score analysis in Figure 7B and C. The deletion also makes the attribution of antigen presentation to the late stages consistent throughout the manuscript.

3. *How confident are the authors that the observed differences in pathways are biologically meaningful? Looking at Figure 7C, the differences in cytokine expression across clusters are not very convincing. In contrast, differences in phagocytosis or antigen presentation across subsets appear more evident.*

Response. We agree with the substance of this and have tempered the interpretation at line 433: "a striking functional dichotomy" now reads "a functional gradient", "the highest scores" now reads "the highest module scores", and "displayed" now reads "showed". The narrower spread of the cytokine module in Figure 7C is expected, since cytokine transcripts are induction dependent and short lived and these cells were profiled directly ex vivo without stimulation; the functional evidence for cytokine production is therefore at protein level, from intracellular staining across eight donors and four stimuli (lines 467 to 486). The phagocytosis and antigen presentation differences are supported both transcriptionally and functionally (Figure 7F). As the single-cell data derive from one donor (Methods line 801), per-cell adjusted p values across clusters would constitute pseudoreplication and were deliberately not reported.

4. *Similar frequencies of TNF-expressing cells are observed in the non-stimulated condition compared with the different stimulation conditions. Moreover, for most stimuli, the frequencies appear to decrease, with the possible exception of Ig + CD40L and perhaps CpG for the CD11c+ subset (Figure 7G). Could the limited effect of stimulation be due to the relatively high frequencies observed in the non-stimulated condition for some individuals?*

Response. We re-examined the per-donor data; the non-stimulated frequencies do not account for the limited apparent effect. As stated at line 472, every condition, including medium only, received a 4 h PMA and ionomycin restimulation before intracellular staining, so the non-stimulated condition is a maximal receptor-independent control rather than a resting baseline, which is why TNF frequencies are already high there. All comparisons are paired within donor, so between-individual variation in the medium-only frequencies cannot drive the differences reported. The apparent decreases are small and not significant, consistent with receptor desensitisation and with cytokine release during the 20 h priming period before the brefeldin A block. No change to the manuscript was made for this point.

5. *Can the authors provide the gating strategy for TNFα- and IL-10-positive cells?*

Response. The gating strategy for TNFα and IL-10 positive cells is already provided in the Appendix, in Appendix Figure S9F and G (previously Appendix Figure 9F and G, renumbered following the editorial request). No new data were required.

6. *For IL-10, stimulation appears to increase the frequency of positive cells only with CpG and R848.*

Response. Agreed. Line 484 now includes ", However, CpG and R848 stimulation led significantly increased IL-10 production compared with NS in Int-ABC2 and Late-ABCs…" before the citation of Figure 7J. This is consistent with endosomal Toll-like receptor signalling driving regulatory IL-10 in human B cells, through TLR9 for CpG and TLR7/8 for R848, whereas BCR and CD40 co-stimulation and TLR2/6 ligation do not.

7. *In Figure 7I, stimulation effects can be observed for some subsets, particularly with Ig + CD40L and CpG. I think an additional plot comparing the non-stimulated condition with each stimulation (rather than comparing frequencies between subsets within each stimulation) would make it clearer when stimulation has an effect.*

Response. Agreed. The requested plot has been added to Appendix Figure S9, in which the non-stimulated condition is compared with each stimulation within each subset, rather than frequencies being compared between subsets within each stimulation. All comparisons are paired within donor and follow the same test and plotting conventions as Figure 7I, and the new panel is cited from the Results alongside Figure 7I. The subset-wise presentation in Figure 7I has been retained, as the two views are complementary.

8. *Please acknowledge as a limitation that sex, a major determinant of immune responses, was not considered in the analyses, and comment on whether sex-related differences might be expected.*

Response. Agreed. The limitations paragraph now states at line 608-612 that sex was not considered as a variable, and comments on the direction in which differences would be expected: ABC accumulation is TLR7 dependent and female biased in mice, and TLR7 is X encoded and partially escapes X chromosome inactivation (PMID 21543762), so a comparable female bias is possible in humans. Such differences would be expected to shift the relative size of the subsets rather than the differentiation structure described here.

9. *In Figure 2F, the lineage numbers appear to be exchanged compared with the description in the Results.*

Response. The figure is correct and the ambiguity was in the text, which distinguished the two trajectories only by ordinals. The Results now name them explicitly: Lineage 1 from switched memory B cells to Early-ABCs, and Lineage 2 from naïve B cells through non-switched memory B cells and Int-ABCs to Late-ABCs, matching Figure 2F. No change to the figure was required.

10. *Line 236: by "was independent of memory," do the authors mean "was independent of exposure"?*

Response. The referee's reading is correct and the suggested wording has been adopted: line 249 now reads "was independent of prior antigen exposure". The later statement on prior malaria exposure was already unambiguous and is unchanged.

11. *The subsection title, "High-resolution transcriptomic and V(D)J sequencing of CD11c+ B Cells reveals additional intermediate ABC states with different plasma cell differentiation potential," should be revised, as the V(D)J sequencing results are presented in the following section.*

Response. Agreed. The title now reads "High-resolution transcriptomic analysis of CD11c+ B Cells reveals additional intermediate ABC states". We also removed the reference to plasma cell differentiation as that data has now been moved to figure 7. Only the heading was changed; the V(D)J results in the following section are unchanged.

Referee #2

The points I had raised in my original report have been adequately addressed.

Response. We thank Referee #2 for the assessment of the previous version.

Editorial requests

All editorial and formatting issues were resolved by the authors.

Dear Chris,

Please accept my apologies for the rather slow re-review process on this occasion and my congratulations on an excellent manuscript. I am very pleased to inform you that referee #1 recognizes that all previous concerns have now been satisfactorily addressed, and your manuscript has now been accepted for publication in The EMBO Journal. Thank you for comprehensively addressing the initially raised referees' concerns and our editorial requests for changes and corrections.

IMPORTANT:
There is only one minor remaining point from our side that must be addressed before we can move forward with the next steps towards the publication of your manuscript: the exact p-values must be provided in the legends of the Figures listed in my previous decision letter. Please send me, as soon as possible, a new version of the main manuscript file by e-mail with these exact p-values included in the Figure legends, as appropriate. Please make sure that no other changes are introduced in the manuscript file at this stage, as it would significantly delay its further processing.

Once we have received this information from you, your manuscript will be processed for publication by EMBO Press. It will be copy edited and you will receive page proofs prior to publication. Please note that you will be contacted by Springer Nature Author Services to complete licensing and payment information.

You may qualify for financial assistance for your publication charges - either via a Springer Nature fully open access agreement or an EMBO initiative. Check your eligibility: https://link.springer.com/journal/44318/how-to-publish-with-us

Should you be planning a Press Release on your article, please notify the production team via this form in order to coordinate publication and release dates: https://forms.cloud.microsoft/e/eH4A8JVP6g

If you have any questions, please do not hesitate to contact the Editorial Office. Thank you for your contribution to The EMBO Journal. Working with you has been a pleasure.

Best regards,

Ioannis

Ioannis Papaioannou, PhD
Senior Editor, The EMBO Journal - EMBO Reports - Molecular Systems Biology
i.papaioannou@embojournal.org

Referee #1:

The authors have satisfactorily addressed all of my remaining comments, and I have no further concerns.

*****
Please note that it is The EMBO Journal policy for the transcript of the editorial process (containing referee reports and your response letters) to be published as an online supplement to each paper. If you should prefer removal of any referee-only figures included in the point-by-point response(s), e.g. because they may still be used for future publication or because they have been reproduced from published work by others, please do let us know immediately via response email.
More information is available here: https://link.springer.com/partners/embo-press/editorial-policies#Peer%20review

** Click here to be directed to your login page: https://emboj.msubmit.net

EMBO Press Author Checklist

Corresponding Author Name: Christopher Sundling	
Journal Submitted to: The EMBO Journal	
Manuscript Number: EMBOJ-2025-121231R	

USEFUL LINKS FOR COMPLETING THIS FORM
The EMBO Journal - Author Guidelines
EMBO Reports - Author Guidelines
Molecular Systems Biology - Author Guidelines
EMBO Molecular Medicine - Author Guidelines

Reporting Checklist for Life Science Articles (updated January
This checklist is adapted from Materials Design Analysis Reporting (MDAR) Checklist for Authors. MDAR establishes a minimum set of requirements in transparent reporting in the life sciences (see Statement of Task: 10.31222/osf.io/9sm4x). Please follow the journal's guidelines in preparing your
Please note that a copy of this checklist will be published alongside your article.

Abridged guidelines for figures
1. Data
The data shown in figures should satisfy the following conditions:
- the data were obtained and processed according to the field's best practice and are presented to reflect the results of the experiments in an accurate and unbiased manner.
- ideally, figure panels should include only measurements that are directly comparable to each other and obtained with the same assay.
- plots include clearly labeled error bars for independent experiments and sample sizes. Unless justified, error bars should not be shown for technical
- if n<5, the individual data points from each experiment should be plotted. Any statistical test employed should be justified.
- Source Data should be included to report the data underlying figures according to the guidelines set out in the authorship guidelines on Data

2. Captions
Each figure caption should contain the following information, for each panel where they are relevant:
- a specification of the experimental system investigated (eg cell line, species name).
- the assay(s) and method(s) used to carry out the reported observations and measurements.
- an explicit mention of the biological and chemical entity(ies) that are being measured.
- an explicit mention of the biological and chemical entity(ies) that are altered/varied/perturbed in a controlled manner.
- the exact sample size (n) for each experimental group/condition, given as a number, not a range;
- a description of the sample collection allowing the reader to understand whether the samples represent technical or biological replicates (including how many animals, litters, cultures, etc.).
- a statement of how many times the experiment shown was independently replicated in the laboratory.
- definitions of statistical methods and measures:
  - common tests, such as t-test (please specify whether paired vs. unpaired), simple χ2 tests, Wilcoxon and Mann-Whitney tests, can be unambiguously identified by name only, but more complex techniques should be described in the methods section;
  - are tests one-sided or two-sided?
  - are there adjustments for multiple comparisons?
  - exact statistical test results, e.g., P values = x but not P values < x;
  - definition of 'center values' as median or average;
  - definition of error bars as s.d. or s.e.m.

Please complete ALL of the questions below.
Select "Not Applicable" only when the requested information is not relevant for your study.

Materials

Newly Created Materials	Information included in the manuscript?	In which section is the information available? (Reagents and Tools Table, Materials and Methods, Figures, Data Availability Section)
New materials and reagents need to be available; do any restrictions apply?	Not Applicable	

Antibodies	Information included in the manuscript?	In which section is the information available? (Reagents and Tools Table, Materials and Methods, Figures, Data Availability Section)
For **antibodies** provide the following information: - Commercial antibodies: RRID (if possible) or supplier name, catalogue number and or/clone number - Non-commercial: RRID or citation	Yes	Reagents and Tools Table

DNA and RNA sequences	Information included in the manuscript?	In which section is the information available? (Reagents and Tools Table, Materials and Methods, Figures, Data Availability Section)
**Short novel DNA or RNA including primers, probes**: provide the sequences.	Not Applicable	

Cell materials	Information included in the manuscript?	In which section is the information available? (Reagents and Tools Table, Materials and Methods, Figures, Data Availability Section)
**Cell lines:** Provide species information, strain. Provide accession number in repository **OR** supplier name, catalog number, clone number, and/**OR** RRID.	Yes	Reagents and Tools Table
**Primary cultures:** Provide species, strain, sex of origin, genetic modification status.	Yes	Material and Method
Report if the cell lines were recently **authenticated** (e.g., by STR profiling) and tested for mycoplasma contamination.	Not Applicable	

Experimental animals	Information included in the manuscript?	In which section is the information available? (Reagents and Tools Table, Materials and Methods, Figures, Data Availability Section)
**Laboratory animals or Model organisms:** Provide species, strain, sex, age, genetic modification status. Provide accession number in repository **OR** supplier name, catalog number, clone number, **OR** RRID.	Not Applicable	
**Animal observed in or captured from the field:** Provide species, sex, and age where possible.	Not Applicable	
Please detail **housing and husbandry conditions**.	Not Applicable	

Plants and microbes	Information included in the manuscript?	In which section is the information available? (Reagents and Tools Table, Materials and Methods, Figures, Data Availability Section)
**Plants:** provide species and strain, ecotype and cultivar where relevant, unique accession number if available, and source (including location for collected wild specimens).	Not Applicable	
**Microbes:** provide species and strain, unique accession number if available, and source.	Not Applicable	

Human research participants	Information included in the manuscript?	In which section is the information available? (Reagents and Tools Table, Materials and Methods, Figures, Data Availability Section)
If collected and within the bounds of privacy constraints report on age, sex and gender or ethnicity for all study participants.	Yes	Material and Method, Supplementary Table

Core facilities	Information included in the manuscript?	In which section is the information available? (Reagents and Tools Table, Materials and Methods, Figures, Data Availability Section)
If your work benefited from core facilities, was their service mentioned in the acknowledgments section?	Yes	Acknowledgements

Design

Study protocol	Information included in the manuscript?	In which section is the information available? (Reagents and Tools Table, Materials and Methods, Figures, Data Availability Section)
If study protocol has been **pre-registered, provide DOI in the manuscript**. For clinical trials, provide the trial registration number **OR** cite DOI.	Not Applicable	
Report the **clinical trial registration number** (at ClinicalTrials.gov or equivalent), where applicable.	Not Applicable	

Laboratory protocol	Information included in the manuscript?	In which section is the information available? (Reagents and Tools Table, Materials and Methods, Figures, Data Availability Section)
Provide DOI OR other citation details if **external detailed step-by-step protocols** are available.	Not Applicable	

Experimental study design and statistics	Information included in the manuscript?	In which section is the information available? (Reagents and Tools Table, Materials and Methods, Figures, Data Availability Section)
Include a statement about **sample size** estimate even if no statistical methods were used.	Yes	Figures
Were any steps taken to minimize the effects of subjective bias when allocating animals/samples to treatment (e.g. **randomization procedure**)? If yes, have they been described?	Not Applicable	
Include a statement about **blinding** even if no blinding was done.	Not Applicable	
Describe **inclusion/exclusion criteria** if samples or animals were excluded from the analysis. Were the criteria pre-established?<br><br>If sample or data points were omitted from analysis, report if this was due to attrition or intentional exclusion and provide justification.	Not Applicable	
For every figure, are **statistical tests** justified as appropriate? Do the data meet the assumptions of the tests (e.g., normal distribution)? Describe any methods used to assess it. Is there an estimate of variation within each group of data? Is the variance similar between the groups that are being statistically compared?	Yes	Result (Figure legend)

Sample definition and in-laboratory replication	Information included in the manuscript?	In which section is the information available? (Reagents and Tools Table, Materials and Methods, Figures, Data Availability Section)
In the figure legends: state number of times the experiment was **replicated** in laboratory.	Yes	Figures Legend
In the figure legends: define whether data describe **technical or biological replicates**.	Not Applicable	

Ethics

Ethics	Information included in the manuscript?	In which section is the information available? (Reagents and Tools Table, Materials and Methods, Figures, Data Availability Section)
Studies involving **human participants**: State details of **authority granting ethics approval** (IRB or equivalent committee(s), provide reference number for approval.	Yes	Material and Method
Studies involving **human participants**: Include a statement confirming that **informed consent** was obtained from all subjects and that the experiments conformed to the principles set out in the WMA Declaration of Helsinki and the Department of Health and Human Services Belmont Report.	Yes	Material and Method
Studies involving **human participants:** For publication of **patient photos**, include a statement confirming that consent to publish was obtained.	Not Applicable	
Studies involving experimental **animals**: State details of **authority granting ethics approval** (IRB or equivalent committee(s), provide reference number for approval. Include a statement of compliance with ethical regulations.	Not Applicable	
Studies involving **specimen and field samples:** State if relevant **permits** obtained, provide details of authority approving study; if none were required, explain why.	Not Applicable	

Dual Use Research of Concern (DURC)	Information included in the manuscript?	In which section is the information available? (Reagents and Tools Table, Materials and Methods, Figures, Data Availability Section)
Could your study fall under dual use research restrictions? Please check biosecurity documents and list of **select agents and toxins** (CDC): https://www.selectagents.gov/sat/list.htm	Not Applicable	
If you used a select agent, is the security level of the lab appropriate and reported in the manuscript?	Not Applicable	
If a study is subject to dual use research of concern regulations, is the name of the **authority granting approval and reference number** for the regulatory approval provided in the manuscript?	Not Applicable	

Reporting

The MDAR framework recommends adoption of discipline-specific guidelines, established and endorsed through community initiatives. Journals have their own policy about requiring specific guidelines and recommendations to complement MDAR.

Adherence to community standards	Information included in the manuscript?	In which section is the information available? (Reagents and Tools Table, Materials and Methods, Figures, Data Availability Section)
State if relevant guidelines or checklists (e.g., **ICMJE, MIBBI, ARRIVE, PRISMA**) have been followed or provided.	Not Applicable	
For **tumor marker prognostic studies,** we recommend that you follow the **REMARK** reporting guidelines (see link list at top right). See author guidelines, under 'Reporting Guidelines'. Please confirm you have followed these guidelines.	Not Applicable	
For **phase II and III randomized controlled trials**, please refer to the **CONSORT** flow diagram (see link list at top right) and submit the CONSORT checklist (see link list at top right) with your submission. See author guidelines, under 'Reporting Guidelines'. Please confirm you have submitted this list.	Not Applicable	

Data Availability

Data availability	Information included in the manuscript?	In which section is the information available? (Reagents and Tools Table, Materials and Methods, Figures, Data Availability Section)
Have **primary datasets** been deposited according to the journal's guidelines (see 'Data Deposition' section) and the respective accession numbers provided in the Data Availability Section?	Yes	Data availability section
Were **human clinical and genomic datasets** deposited in a public access-controlled repository in accordance to ethical obligations to the patients and to the applicable consent agreement?	Yes	Data availability section
Are **computational models** that are central and integral to a study available without restrictions in a machine-readable form? Were the relevant accession numbers or links provided?	Yes	Code used for data analysis are provided at the repository and via the lab github page with a link in the Data availabiilty section
If publicly available data were reused, provide the respective **data citations in the reference list**.	Yes	We include references to the papers where the original data were generated
